# Bacteria-Killing Type IV Secretion Systems

**DOI:** 10.3389/fmicb.2019.01078

**Published:** 2019-05-21

**Authors:** Germán G. Sgro, Gabriel U. Oka, Diorge P. Souza, William Cenens, Ethel Bayer-Santos, Bruno Y. Matsuyama, Natalia F. Bueno, Thiago Rodrigo dos Santos, Cristina E. Alvarez-Martinez, Roberto K. Salinas, Chuck S. Farah

**Affiliations:** ^1^Departamento de Bioquímica, Instituto de Química, Universidade de São Paulo, São Paulo, Brazil; ^2^Departamento de Genética, Evolução, Microbiologia e Imunologia, Instituto de Biologia, University of Campinas (UNICAMP), Campinas, Brazil

**Keywords:** bacterial competition, Xanthomonadales, type IV immunity protein, type IV secretion effector, type IV secretion system, X-Tfe, X-Tfi, X-T4SS

## Abstract

Bacteria have been constantly competing for nutrients and space for billions of years. During this time, they have evolved many different molecular mechanisms by which to secrete proteinaceous effectors in order to manipulate and often kill rival bacterial and eukaryotic cells. These processes often employ large multimeric transmembrane nanomachines that have been classified as types I–IX secretion systems. One of the most evolutionarily versatile are the Type IV secretion systems (T4SSs), which have been shown to be able to secrete macromolecules directly into both eukaryotic and prokaryotic cells. Until recently, examples of T4SS-mediated macromolecule transfer from one bacterium to another was restricted to protein-DNA complexes during bacterial conjugation. This view changed when it was shown by our group that many *Xanthomonas* species carry a T4SS that is specialized to transfer toxic bacterial effectors into rival bacterial cells, resulting in cell death. This review will focus on this special subtype of T4SS by describing its distinguishing features, similar systems in other proteobacterial genomes, and the nature of the effectors secreted by these systems and their cognate inhibitors.

## Introduction

**FIGURE 1:**
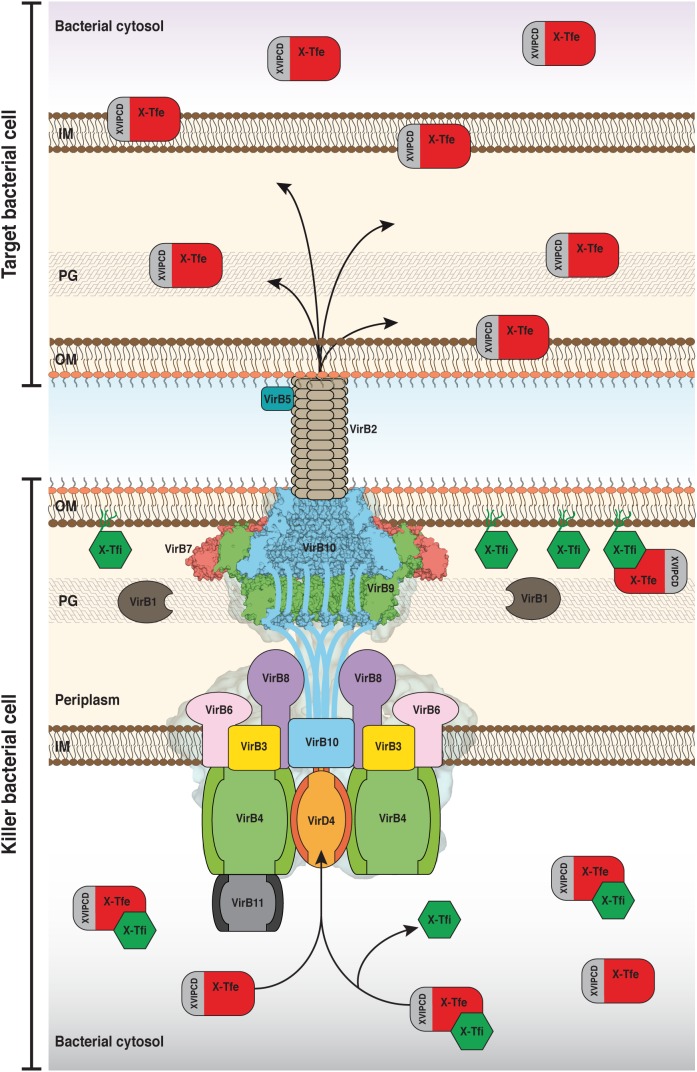
Schematic model of the structure and function of the bacteria-killing Xanthomonadales-like Type IV secretion systems (X-T4SSs). The model shows the interface between two bacterial cells. The killer cell (below) is armed with an X-T4SS whose general architecture is based on the negative-stained electron microscope map of the R388 T4SS shown in the background (Low et al., 2014; Redzej et al., 2017) and the cryo-EM structure of the *X. citri* core complex (VirB7, VirB9, and VirB10; Sgro et al., 2018) associated with the outer membrane (OM). The disordered N-terminal domains of the VirB10 subunits extend down from the core complex and pass through the inner membrane. The inner membrane (IM) complex is made up of VirB3, VirB6, VirB8, the three ATPases VirB4, VirB11, and VirD4 as well as the aforementioned N-terminal segments of VirB10. Pili, made up of VirB2 and VirB5, mediate intercellular contacts. X-T4SS effectors (X-Tfes) interact, via their XVIPCD domains, with VirD4 and are subsequently transferred to the T4SS for translocation into the target cell where they will degrade target structures such as membrane phospholipids or carbohydrate and peptide linkages in the peptidoglycan (PG) layer. Prior to secretion, X-Tfes whose activities could target cytosolic substrates can be inhibited by cytosolic variants of their cognate immunity proteins (X-Tfis). If X-Tfes make their way into the periplasm, either by leakage from the secretion channel or by injection by neighboring cells of the same species, they will be inhibited by the periplasmic lipoprotein forms of the cognate X-Tfi. Portions of the Figure were adapted from Low et al. (2014) and Sgro et al. (2018) with permission from the publishers.

Type IV secretion systems (T4SSs) have been studied since the birth of modern molecular biology, starting with the description of bacterial conjugation over 70 years ago (Lederberg and Tatum, 1946). It quickly became evident that the T4SS-mediated horizontal transfer of genetic material is a major contributor to bacterial evolution, making it necessary to consider lateral connections between lineages for a complete description of the bacterial tree of life (de la Cruz and Davies, 2000). Horizontal gene transfer is also one of the principal mechanisms for the spread of genes conferring resistance to antibiotics (Cabezon et al., 2015). Moreover, many pathogenic bacteria use T4SSs to facilitate their proliferation and survival inside eukaryotic hosts, typically by the secretion of protein effectors or protein-DNA complexes (Gonzalez-Rivera et al., 2016). T4SSs are thus important virulence factors in a variety of human diseases, including whooping cough (*Bordetella pertussis*; Locht et al., 2011; Carbonetti, 2015), cat-scratch fever (*Bartonella henselae*; Siamer and Dehio, 2015), brucellosis (*Brucella* spp.; Ke et al., 2015), Legionnaire’s pneumonia (*Legionella pneumophila*; Finsel and Hilbi, 2015), Q fever (*Coxiella burnetii*; Moffatt et al., 2015) and peptic ulcer and gastric cancer (*Helicobacter pylori*; Naumann et al., 2017). One of the most well-characterized T4SSs is that of *Agrobacterium tumefaciens* which injects nucleoprotein complexes and protein factors into plant cells (Alvarez-Martinez and Christie, 2009; Li and Christie, 2018). Furthermore, specialized T4SSs from *Neisseria gonorrhoeae* or *H. pylori* secrete DNA to the extracellular milieu or uptake DNA from the environment to the bacterial cytoplasm, respectively (Hofreuter et al., 2001; Hamilton et al., 2005; Callaghan et al., 2017). Finally, the plant pathogen *Xanthomonas citri* (Oliveira et al., 2016; Sgro et al., 2018; Souza et al., 2015) and, more recently, the opportunistic human pathogen *Stenotrophomonas maltophilia* (preprint: Bayer-Santos et al., 2019), have been shown to use a T4SS to inject toxic effectors into target bacteria, thus inducing the death of rival cells (Figure 1).

T4SSs are structurally very diverse. For example, the related pKM101 and R388 plasmid-encoded conjugation systems (Chandran et al., 2009; Fronzes et al., 2009; Rivera-Calzada et al., 2013) and the pathogenic *Legionella* Dot/Icm (Ghosal et al., 2017; Chetrit et al., 2018) and *H. pylori* Cag (Frick-Cheng et al., 2016; Chang et al., 2018) effector-secreting systems, while all exhibiting an outer membrane-associated core complex with 14-fold or 13-fold symmetry, present significantly different features in terms of their overall size. These systems also display a varied set of both functional and structural subunits, and even the homologous subunits have very low sequence similarity and frequently present modified domain architectures (Alvarez-Martinez and Christie, 2009; Christie et al., 2014; Guglielmini et al., 2014; Christie, 2016; Grohmann et al., 2017). For these reasons, the T4SSs from Gram-negative bacteria have been divided into two major classes, denoted A and B (Christie and Vogel, 2000), and classification systems based on detailed phylogenetic analysis have divided Gram-negative and Gram-positive T4SSs into up to 8 classes (Guglielmini et al., 2014).

The canonical class A, best represented by the *A. tumefaciens vir* system and those coded by conjugative plasmids pKM101, R388, and RP4, have the basic set of 12 conserved subunits, named VirB1 to VirB11 plus VirD4 (Tzfira and Citovsky, 2006). The overall organization of the canonical class A T4SSs has been revealed in electron microscopy studies (Low et al., 2014; Redzej et al., 2017) and can be divided into two general (sub)complexes (Figure 1). The inner membrane complex is made up of subunits embedded in, or associated with, the inner membrane: VirB3, VirB4, VirB6, VirB8, VirB11, and VirD4. The outer membrane or “core” complex is comprised of the subunits VirB7, VirB9, and VirB10. These two complexes are connected by a flexible “stalk” of unknown composition, though it has been proposed to be made up, at least in part, by the disordered N-terminal domain of VirB10 (which also has an N-terminal transmembrane helix embedded in the inner membrane) and/or the C-terminal domain of VirB8 (Christie, 2016; Waksman, 2019). In addition to this transmembrane structure, there are extracellular pili made of subunits VirB2 and VirB5, that are presumably involved in making contact with the membrane of the target cell or organelle (Alvarez-Martinez and Christie, 2009). Even within the class A T4SSs, a large degree of sequence and size diversity has been observed for many of the subunits in different species. This is perhaps most starkly exemplified when considering the *H. pylori* Cag T4SS which, in addition to orthologs of the basic set of canonical class A subunits, possesses another five subunits that are required for proper function (Backert et al., 2015; Frick-Cheng et al., 2016).

The even more distantly related class B includes T4SSs found in the pathogens *L. pneumophila*, *C. burnetii*, and *Rickettsiella grylli* as well as in the IncI conjugative plasmids R64 and ColIb-P9 (Sexton and Vogel, 2002). *L. pneumophila* causes Legionnaire’s disease in humans by infecting alveolar macrophages where it replicates within a specialized vacuole (Backert and Meyer, 2006; Ensminger and Isberg, 2009). Its Dot/Icm T4SS is made up of 27 components and secretes more than 300 effector proteins that manipulate signal transduction pathways in the host cell, primarily affecting organelle trafficking (Hilbi et al., 2017; Qiu and Luo, 2017). The bacteria-killing T4SSs, which is the topic of this review, belong to the canonical class A T4SSs, although they do have some structurally distinguishing features as described below.

The Xanthomonadales order of Gammaproteobacteria (Saddler and Bradbury, 2007), recently divided into two orders, Xanthomonadales (families *Xanthomonadaceae* and *Rhodanobacteraceae*) and Nevskiales (Naushad et al., 2015), include several hundred phytopathogenic species of the genera *Xanthomonas* and *Xylella* as well as important and ubiquitous soil, water and plant-associated bacteria of the genera *Stenotrophomonas*, *Lysobacter*, *Luteimonas*, *Pseudoxanthomonas*, *Rhodanobacter*, *Luteibacter*, *Dyella*, *Frateuria*, *Aquimonas*, and others (Van Sluys et al., 2002; Saddler and Bradbury, 2007; Looney et al., 2009; Mansfield et al., 2012). Some *Stenotrophomonas* strains are opportunistic pathogens of immunosuppressed human patients (Chang et al., 2015) and some *Stenotrophomonas* and *Lysobacter* strains have been recognized as potential biological control agents in combating plant diseases caused by fungi or other bacteria (Hayward et al., 2010; Mukherjee and Roy, 2016; Panthee et al., 2016). Other species from the genera *Lysobacter* and *Luteimonas* have been isolated from extreme environments (Brito et al., 2013; Zhang et al., 2015). Although the role of types II and III secretion systems in the virulence of species of the genus *Xanthomonas* is already well established (Buttner and Bonas, 2010), until a few years ago there was little information available on the functions of other secretion systems in these bacterial species. An accompanying article in this series deals with the recently discovered role of the *X. citri* Type VI secretion system (T6SS) in protection against predation by phagocytic amoebas (Bayer-Santos et al., 2018). In this review, we will focus on the special characteristics of the Xanthomonadales Type IV secretion systems, first described in *X. citri*, and their role in the contact-dependent killing of rival Gram-negative species. The review will focus on describing the distinguishing structural features of the T4SS components encoded by the chromosomal *virB* locus of *X. citri*, their conservation in homologous systems in the order Xanthomonadales and other proteobacterial genomes, the nature of the effectors secreted by these systems and the cognate inhibitors of these effectors.

## The Chromosomally Coded T4SS of *Xanthomonas citri*

**FIGURE 2:**
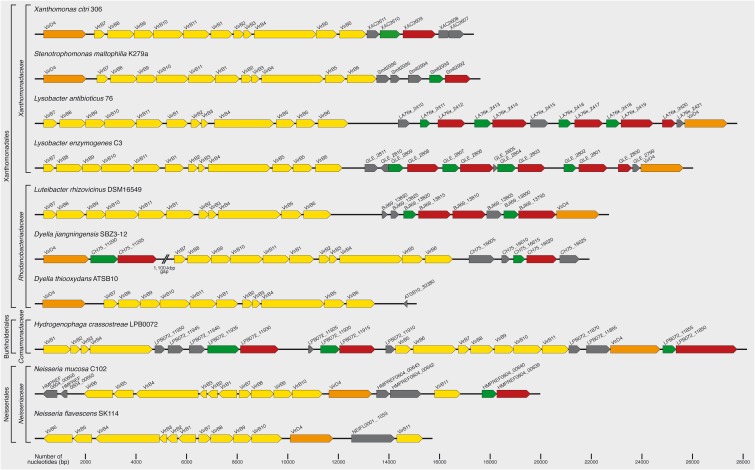
*Xanthomonas citri* chromosomal *vir* locus and its homologs in other species. The top line presents the T4SS encoded by the chromosomal *vir* locus of *X. citri* 306 (Da Silva et al., 2002; Alegria et al., 2005). It contains the canonical set of 12 components found in other class A T4SSs. Genes coding for T4SSs with similar characteristics to that of *X. citri* can be identified in the chromosomes of many other species (see Table 1 for an extensive list). Shown here are representative examples from *Stenotrophomonas maltophilia* K279a (Crossman et al., 2008), *Lysobacter antibioticus* 76 (de Bruijn et al., 2015), *Lysobacter enzymogenes* C3 (unpublished; GenBank accession CP013140), *Luteibacter rhizovicinus* DSM16549 (unpublished; GenBank accession CP017480), *Dyella jiangningensis* SBZ3-12 (Bao et al., 2014), *Dyella thiooxydans* ATSB10 (unpublished; GenBank accession CP014841), *Hydrogenophaga crassostreae* LPB0072 (unpublished; GenBank accession LVWD01000013), *Neisseria mucosa* C102 (unpublished, GenBank accession GCA_000186165) and *Neisseria flavescens* SK114 (unpublished; GenBank accession ACQV01000009). VirB and VirD4 genes are shown in yellow and orange, respectively. Xanthomonadales-like T4SS effectors (X-Tfes) and immunity proteins (X-Tfis) are colored red and green, respectively. Other open reading frames coding for proteins of unknown function are shown in gray.

The T4SS encoded by the chromosomal *vir* locus of *X. citri* contains the canonical set of 12 structural components found in other class A T4SSs (Figure 2; Alegria et al., 2005; Souza et al., 2011). The presence of chromosomally encoded homologs in several other *Xanthomonas* species (see below) suggested an important function in *Xanthomonas* biology. A role in bacterial conjugation or nucleic acid transfer was deemed unlikely since the chromosomal *virB* locus does not contain genes coding for homologs of the DNA processing components of the relaxosome or characteristic palindromic *oriT* sites (Alegria et al., 2005). Furthermore, a knockout of the *virB7* gene in *X. citri* did not affect the development of canker symptoms in citrus plants (Souza et al., 2011) and the deletion of a large part of the homologous operon in *Xanthomonas campestris* pv. *campestris* 8004 did not modify the phenotype of infection in several plants of the *Brassicaceae* family (He et al., 2007), ruling out a direct involvement of the T4SS in *Xanthomonas* virulence (at least in these two species). Our group subsequently demonstrated that this secretion system confers to *X. citri* the capacity to kill other Gram-negative cells in a contact-dependent manner (Souza et al., 2015). The first bacterial killing experiments were performed by confronting *X. citri* with common laboratory strains of *Escherichia coli* as well as the Betaproteobacterium *Chromobacterium violaceum* (Souza et al., 2015) and subsequent experiments demonstrated similar T4SS-dependent killing of several other Gram-negative bacteria but not Gram-positive bacteria (DPS, GUO, WC, and CSF; unpublished). Recently, a CPRG-based colorimetric assay has been employed to monitor the real time kinetics of T4SS-dependent bacterial killing by both *X. citri* (Sgro et al., 2018) and *S. maltophilia* (Preprint: Bayer-Santos et al., 2019). Time-lapse microscopy clearly showed that bacterial killing by *X. citri* and *S. maltophilia* requires cell-cell contact and that the death of target cells is evidenced by the loss of cell turgor and contents over a very short period of time (Souza et al., 2015; Preprint: Bayer-Santos et al., 2019). These T4SSs share with some T6SSs the ability to transfer their toxic effectors directly into rival bacterial species of different orders and phyla and so are important factors for interspecies competition. In this sense, they differ from contact-dependent growth inhibition (CDI) systems (Hayes et al., 2014) and the *Staphylococcus aureus* Type VII secretion system (T7SS; Cao et al., 2016) that seem to be important for competition between cells of the same or closely related species (intraspecies competition).

## Identification of Homologous Systems in the Order Xanthomonadales and Other Proteobacterial Genomes

Several of the *X. citri* T4SS components have some interesting features that distinguish them from their homologs in other more distantly related class A T4SSs involved in horizontal transfer of genetic material. For example, the VirB7 and VirB8 subunits have C-terminal extensions absent in most of their more distantly related homologs (see below). Genes coding for T4SSs with similar characteristics to that of *X. citri* can be identified in the chromosomes of many other *Xanthomonas* species (Figure 2 and Table 1), for example *Xanthomonas campestris* pv. *campestris* B100 (Vorholter et al., 2008), *Xanthomonas albilineans* GPEPC73 (Pieretti et al., 2009), *X. campestris* pv. *vasculorum* NCPPB702 (Studholme et al., 2010) and *X. campestris* pv. *musacearum* NCPPB2005 (Wasukira et al., 2012). The corresponding locus is fragmented in *X. campestris* pv. *campestris* strains ATCC33913 (Da Silva et al., 2002) and 8004 (Qian et al., 2005), with the *virB5* and *virB6* genes found in other regions of the genomes. *X. campestris* pv. *vesicatoria* 85-10 (Thieme et al., 2005) lacks a significant part of the *vir* locus (all that remains is the 5′ region coding for VirD4, VirB7, VirB8, and VirB9). The system is also absent in *Xanthomonas oryzae* strains KACC10331 (Lee et al., 2005), MAFF311018 (Ochiai et al., 2005), PXO99A (Salzberg et al., 2008), and BLS256 (Salzberg et al., 2007), in *Xanthomonas fuscans* subsp. *aurantifolii* strains 10535 and 11122 (Moreira et al., 2010) and in all *Xylella* species sequenced to date. Homologous loci can be found in some Xanthomonadales species of the genera *Stenotrophomonas*, *Pseudoxanthomonas*, *Luteimonas*, *Lysobacter*, *Thermomonas*, *Rhodanobacter*, *Dyella*, *Frateuria*, and *Luteibacter* (Table 1). Interestingly, homologous systems are also found in some species of the Betaproteobacteria orders Burkholderiales (genera *Hydrogenophaga*, *Variovorax*) and Neisseriales (genera *Neisseria* and *Morococcus*) (Table 1). This is consistent with the observation that in some phylogenetic analyses, Xanthomonadales species are observed to branch anomalously with Betaproteobacteria, most probably due to horizontal gene transfer events (Martins-Pinheiro et al., 2004; Comas et al., 2006; Naushad and Gupta, 2013). We will therefore employ the term X-T4SS to designate all Xanthomonadales-like Type IV secretion systems.

**Table 1:** Bacterial strains that code for a putative X-T4SS and X-Tfes substrates.

Organism	Accession^#^	Organism	Accession^#^
***Xanthomonadaceae***			
*Luteimonas* sp. 83-4	NZ_CP029556.1	*Xanthomonas axonopodis* pv. *citrumelo* F1	CP002914.1
***Lysobacter antibioticus* 76**	NZ_CP011129.1	*Xanthomonas axonopodis* pv. *melhusii* LMG9050	LOJW01000012.1
*Lysobacter capsici* AZ78	JAJA02000001.1	*Xanthomonas axonopodis* pv. *vasculorum* NCPPB900	KN173625.1
***Lysobacter enzymogenes* C3**	CP013140.1	*Xanthomonas axonopodis* Xac29-1	CP004399.1
*Lysobacter gummosus* 3.2.11	CP011131.1	*Xanthomonas campestris* pv. *campestris* B100	NC_010688.1
*Lysobacter maris* HZ9B	CP029843.1	*Xanthomonas campestris* pv. *campestris* str. 8004	CP000050.1
*Lysobacter silvestris* AM20-91	NZ_NPZB01000002.1	*Xanthomonas campestris* pv. *campestris* str. ATCC33913	NC_003902.1
*Lysobacter* sp. 4284/11	NZ_RFLY01000011.1	*Xanthomonas campestris* pv. *musacearum* NCPPB2005	AKBE01000043.1
*Lysobacter* sp. cf310	FOSS01000003.1	*Xanthomonas campestris* pv. *raphani* 756C	CP002789.1
*Lysobacter* sp. Root494	NZ_LMFH01000005.1	*Xanthomonas campestris* pv. *vitiscarnosae* LMG939	LOKI01000031.1
*Lysobacter* sp. Root604	LMGS01000001.1	*Xanthomonas campestris* pv. *durantae* LMG696	LOKP01000030.1
*Lysobacter* sp. TY2-98	NZ_CP031413.1	*Xanthomonas cannabis* pv. *cannabis* NCPPB3753	JSZF01000027.1
*Lysobacter* sp. yr284	FNPT01000001.1	***Xanthomonas citri* 306**	AE008923.1
*Lysobacter* sp. zong2l5	NZ_QTSU01000001.1	*Xanthomonas citri* pv. *fuscans* str. 4834-R	FO681494.1
*[Pseudomonas] geniculata* N1	AJLO02000016.1	*Xanthomonas citri* pv. *glycines* str. 8ra	JDSU01000002.1
*Pseudoxanthomonas* sp. CF125	NZ_FNLD01000002.1	*Xanthomonas citri* pv. *malvacearum* MSCT	CP017020.1
*Pseudoxanthomonas* sp. GM95	FOAX01000001.1	*Xanthomonas citri* pv. *phaseoli* var. *fuscans*	CP020979.1
*Pseudoxanthomonas* sp. KAs_5_3	PKSO01000004.1	*Xanthomonas citri* pv. *vignicola* CFBP7113	CP022270.1
*Pseudoxanthomonas spadix* DSM18855	NZ_RDQN01000004.1	*Xanthomonas codiaei* CFBP4690	NZ_MDEC01000025.1
*Pseudoxanthomonas suwonensis* S2	QFNC01000012.1	*Xanthomonas cynarae* CFBP4188	NZ_MDFM01000055.1
*Pseudoxanthomonas wuyuanensis* CGMCC1.10978	NZ_OCND01000011.1	*Xanthomonas dyei* CFBP7245	NZ_MDEE01000016.1
*Stenotrophomonas chelatiphaga* DSM21508	NZ_LDJK01000096.1	*Xanthomonas euvesicatoria* 66b	JSZG01000018.1
*Stenotrophomonas daejeonensis* JCM16244	NZ_LDJP01000037.1	*Xanthomonas euvesicatoria* pv. *citrumelonis* CFBP3371	MDCC01000019.1
*Stenotrophomonas ginsengisoli* DSM24757	NZ_LDJM01000025.1	*Xanthomonas floridensis* WHRI8848	NZ_LXNG01000007.1
*Stenotrophomonas indicatrix* WS40	NZ_PEJS01000001.1	*Xanthomonas fragariae* PD5205	LT853885.1
*Stenotrophomonas koreensis* DSM17805	NZ_LDJH01000013.1	*Xanthomonas gardneri* ATCC19865	AEQX01000084.1
*Stenotrophomonas lactitubi* M15	NZ_PHQX01000001.1	*Xanthomonas hortorum* pv. *hederae* CFBP4925	MDEF01000040.1
*Stenotrophomonas maltophilia* Ab55555	JH791780.1	*Xanthomonas hyacinthi* CFBP1156	NZ_MDEG01000039.1
***Stenotrophomonas maltophilia* K279a**	NC_010943.1	*Xanthomonas hyacinthi* DSM19077	JPLD01000728.1
*Stenotrophomonas maltophilia* MF89	ATAP01000112.1	*Xanthomonas melonis* CFBP4644	MDEH01000006.1
*Stenotrophomonas maltophilia* R551-3	CP001111.1	*Xanthomonas nasturtii* WHRI8853	NZ_LYMI01000032.1
*Stenotrophomonas maltophilia* RA8	CALM01000806.1	*Xanthomonas perforans* Xp4-20	JZUZ01000033.1
*Stenotrophomonas maltophilia* WJ66	KN849114.1	*Xanthomonas phaseoli* pv. *dieffenbachiae* LMG25940	JPYI02000041.1
*Stenotrophomonas pavanii* DSM25135	LDJN01000042.1	*Xanthomonas phaseoli* pv. *manihotis* LMG784	LKKP01000141.1
*Stenotrophomonas rhizophila* OG2	NZ_NCWX01000002.1	*Xanthomonas phaseoli* pv. *phaseoli* NCPPB1158	JSBS02000062.1
*Stenotrophomonas* sp. 92mfcol6.1	FWEU01000001.1	*Xanthomonas pisi* CFBP4643	NZ_MDEI01000004.1
*Stenotrophomonas* sp. AG209	QXDB01000001.1	*Xanthomonas prunicola* CFBP8355	NZ_PHKX01000001.1
*Stenotrophomonas* sp. CC120222-04	FZQI01000004.1	*Xanthomonas sacchari* R1	NZ_CP010409.1
*Stenotrophomonas* sp. BIIR7	NZ_MKCZ01000002.1	*Xanthomonas* sp. CFBP7698	PRDN01000001.1
*Thermomonas fusca* DSM15424	NZ_AUIV01000008.1	*Xanthomonas* sp. CFBP7912	MDSN01000006.1
*Xanthomonas albilineans* GPEPC73	FP565176.1	*Xanthomonas* sp. Leaf131	LMOG01000019.1
*Xanthomonas alfalfae* GEV-Rose-07	MIKD01000086.1	*Xanthomonas* sp. Leaf148	LMOP01000008.1
*Xanthomonas arboricola* pv. *arracaciae* CFBP7407	MIGU01000001.1	*Xanthomonas* sp. NCPPB1128	NZ_LFME01000006.1
*Xanthomonas arboricola* pv. *corylina* CFBP2565	MDSJ01000034.1	*Xanthomonas theicola* CFBP4691	NZ_MIGX01000080.1
*Xanthomonas arboricola* pv. *fragariae* CFBP6773	OEQD01000019.1	*Xanthomonas translucens* pv. *arrhenatheri* LMG727	CXOI01000012.1
*Xanthomonas arboricola* pv. *guizotiae* CFBP7408	MDSK01000048.1	*Xanthomonas translucens* pv. *phlei* LMG730	CXOJ01000010.1
*Xanthomonas arboricola* pv. *juglandis* Xaj417	CP012251.1	*Xanthomonas translucens* pv. *poae* B99	LWSU01000053.1
*Xanthomonas arboricola* pv. *populi* CFBP 3122	MIGV01000035.1	*Xanthomonas translucens* pv. *translucens* DSM18974	CAPJ01000354.1
*Xanthomonas arboricola* pv. *pruni* MAFF301420	BAVC01000014.1	*Xanthomonas vasicola* NCPPB1060	CP034649.1
*Xanthomonas arboricola* pv. *pruni* str. MAFF311562	BAVB01000420.1	*Xanthomonas vasicola* pv. *vasculorum* NCPPB702	NZ_GG699328.2
*Xanthomonas axonopodis* pv. *begoniae* CFBP2524	MPSX01000020.1	*Xanthomonas vesicatoria* ATCC35937	AEQV01000175.1
***Rhodanobacteraceae***			
***Dyella jiangningensis* SBZ3-12**	CP007444.1	*Luteibacter* sp. 329MFSha	NZ_FOJE01000001.1
*Dyella marensis* UNC178MFTsu3.1	FONH01000012.1	*Luteibacter* sp. OK325	QAOX01000004.1
*Dyella* sp. 333MFSha	FNBR01000009.1	*Luteibacter* sp. UNCMF331Sha3.1	FOBU01000002.1
*Dyella* sp. 4MSK11	NZ_QRBF01000004.1	*Luteibacter* sp. UNCMF366Tsu5.1	FPIS01000002.1
*Dyella* sp. AD56	NRDP01000015.1	*Luteibacter yeojuensis* SU11	JZRB01000062.1
*Dyella* sp. AtDHG13	NZ_QICJ01000003.1	*Rhodanobacter fulvus* Jip2	NZ_AJXU01000014.1
*Dyella* sp. OK004	FOZI01000001.1	*Rhodanobacter* sp. 67-28	MKTU01000080.1
***Dyella thiooxydans* ATSB10**	CP014841.1	*Rhodanobacter* sp. C01	NZ_MUNR01000011.1
*Frateuria terrea* CGMCC1.7053	FOXL01000004.1	*Rhodanobacter* sp. C06	NZ_MUNP01000039.1
***Luteibacter rhizovicinus* DSM16549**	CP017480.1	*Rhodanobacter* sp. OK091	FRCH01000001.1
*Luteibacter* sp. 22Crub2.1	FUYT01000025.1	*Rhodanobacter* sp. Root627	NZ_LMGW01000002.1
***Neisseriaceae***			
*Morococcus cerebrosus* CIP81.93	NZ_JUFZ01000115.1	*Neisseria* sp. HMSC069H12	NZ_KV810249.1
*Neisseria dentiae* NCTC13012	NZ_UGQR01000001.1	*Neisseria* sp. HMSC06F02	KQ001448.1
***Neisseria flavescens* SK114**	ACQV01000009.1	*Neisseria* sp. HMSC070A01	LTII01000031.1
***Neisseria mucosa* C102**	GL635793.1	*Neisseria* sp. HMSC071C03	KV836738.1
*Neisseria* sp. HMSC056A03	KV821597.1	*Neisseria* sp. oral taxon 014 str. F0314	GL349413.1
*Neisseria* sp. HMSC064D07	KV831649.1	*Neisseria subflava* C2012011976	NZ_POXP01000001.1
***Comamonadaceae***			
***Hydrogenophaga crassostreae* LPB0072**	LVWD01000013.1	*Hydrogenophaga* sp. H7	MCIC01000014.1
*Hydrogenophaga* sp. A37	MUNZ01000181.1	*Variovorax* sp. Root318D1	NZ_LMCQ01000008.1
**Others**			
*Acinetobacter baumannii* 4300STDY7045681	UFGS01000006.1	*Burkholderiales* bacterium GWF1_66_17	MERT01000006.1
*Bacillus* sp. SRB_336	NZ_NADW01000001.1	*Xanthomonadales* bacterium RIFOXYA1FULL_69_10	MIDV01000097.1
Bacterium AM6	MUYX01000125.1	*Xanthomonadales* bacterium 14-68-21	NCKH01000003.1
*Betaproteobacteria* bacterium HGW	PHCC01000002.1		

*X-T4SSs were identified according to the following criteria: (i) located in chromosomal DNA, (ii) VirB7 subunits have C-terminal N0 domains, (iii) genes for all twelve class A T4SS subunits are present, and (iv) the genomes carry genes for putative effectors (X-Tfes) with C-terminal XVIPCD required for recognition by X-T4SS VirD4. Species names are organized in alphabetical order within each bacterial family. Species discussed in more detail in the main text are shown in bold. ^#^NCBI Reference Sequences or GenBank accession number.*

Figure 2 presents the organization of genetic loci, homologous to the *X. citri* chromosomal *vir* locus, that are found in the genomes of a few bacterial species selected from genera of the *Xanthomonadaceae* family (*Xanthomonas*, *Stenotrophomonas*, and *Lysobacter*), and *Rhodanobacteraceae* family (*Dyella* and *Luteibacter*) within the order Xanthomonadales. Also shown are examples of genetic loci that code for X-T4SSs found in the more distant Betaproteobacteria genera of the *Comamonadaceae* family (*Hydrogenophaga*) and *Neisseriaceae* family (*Neisseria*). One interesting observation is that the loci in species from the Xanthomonadales order seem to have one operon coding for all 11 VirB components, beginning with the *virB7* gene. On the other hand, in *Hydrogenophaga crassostreae*, the operon has been divided into two segments (*virB7-11* and *virB1-6*) and in *Neisseria mucosa* and *N. flavescens* it has been divided into three or more segments (Figure 2). The positions of the *virD4* genes also vary: in most *Xanthomonas* and *Stenotrophomonas* species it is found immediately upstream of the *virB7* gene while in the more distantly related species it appears upstream, downstream or inserted between segments coding for the *virB* genes (Figure 2).

## Distinguishing Structural Features of the Bacteria-Killing Xanthomonadales-Like T4SSs (X-T4SSs)

Supplementary Figures S1–S12 present multiple amino acid sequence alignments of VirB1–VirB11 and VirD4 (respectively) X-T4SS components coded by the homologous loci presented in Figure 2. What follows in this section is a brief description of some interesting structural features that can be identified from these alignments and, in some cases, their correlations with known structures and site-directed mutagenesis studies. The observations gleaned from these comparisons are likely to apply to most of the X-T4SSs listed in Table 1.

### VirB7, VirB9, and VirB10: Components of the Core Complex

**FIGURE 3:**
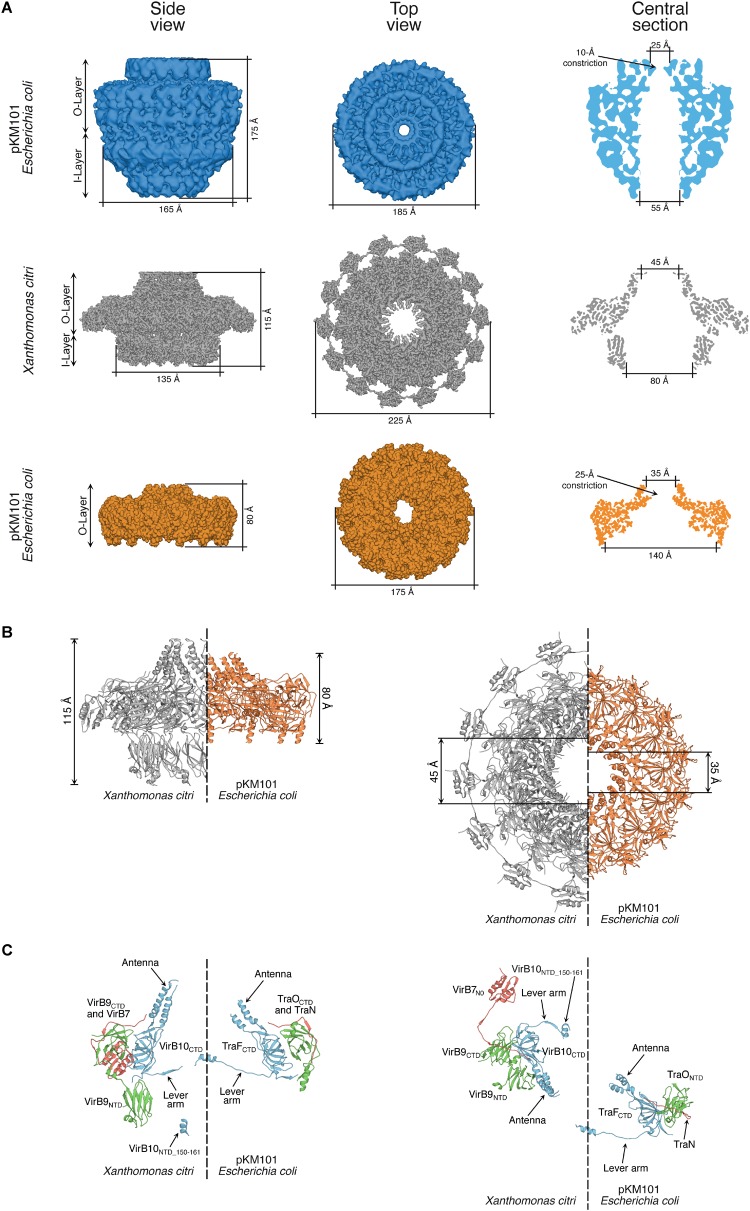
Comparison of core complex structures. **(A)** Comparison of the electron microscopy maps of the full-length core complexes from pKM101 (12.4 Å resolution; top row; Rivera-Calzada et al., 2013) and *X. citri* T4SSs (3.3 Å resolution; middle row; Sgro et al., 2018). Also shown is the electron density map of the O-layer of the pKM101 core complex obtained by X-ray crystallography (2.9 Å resolution; lower row; Chandran et al., 2009). General features and dimensions are shown for side and top views, and for a central section. **(B)** Side-by-side comparison of the atomic models of the *X. citri* core complex (gray) and pKM101 (orange) O-layers. General features and dimensions are shown for side and top views. **(C)** Side-by-side comparison of the atomic models of the VirB7–VirB9–VirB10 trimer and TraN-TraO-TraF trimer in the *X. citri* core complex and pKM101 O-layer, respectively. Colors: VirB10 and TraF (blue), VirB9 and TraO (green), VirB7 and TraN (red). Side (left) and top (right) views are shown of diametrically opposed trimers taken from the side-by-side comparisons shown in **B**. NTD, N-terminal domain; CTD, C-terminal domain. Portions of the Figure were adapted from Rivera-Calzada et al. (2013) and Sgro et al. (2018) with permission from the publishers.

**FIGURE 4:**
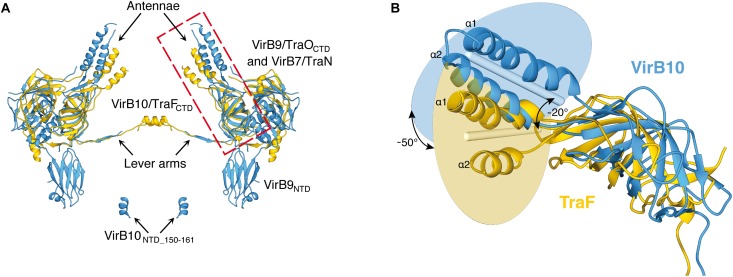
Relative orientations of the antennae that form the outer membrane pore in the *X. citri* and pKM101 core complexes. **(A)** Superposition of the atomic models of diametrically opposed VirB7–VirB9–VirB10 trimers of the *X. citri* core complex (blue) and TraN-TraO-TraF trimers of the pKM101 O-layer (yellow). **(B)** Details of the relative orientations of the VirB10 and TraF C-terminal domains. The structures shown correspond to the red rectangle in **(A)**. The blue and yellow circles represent the planes that contain the central axes of the two antenna helices (α1 and α2). The blue and yellow rods represent the average vector between the two helices in each protein. The angles between the planes (∼50°) and between the rods (∼20°) are shown. Figure derived from Sgro et al. (2018) with modifications. NTD, N-terminal domain; CTD, C-terminal domain. This Figure was adapted from and Sgro et al. (2018) with permission from the publishers.

The 2.9 Å resolution crystal structure of the outer membrane layer of the pKM101 core complex (Chandran et al., 2009) and the recently published 3.3 Å resolution cryo-electron microscopy (cryo-EM) structure of the complete *X. citri* core complex (Sgro et al., 2018), along with the lower resolution EM maps of the complete pKM101 (Fronzes et al., 2009; Rivera-Calzada et al., 2013), R388 (Low et al., 2014) and *A. tumefaciens* (Gordon et al., 2017) core complexes have provided us with the greatest detail as yet of the periplasmic channel that connects the inner and outer membranes of class A T4SSs. These structures, are all made of 14 copies of VirB7–VirB9–VirB10 heterotrimers (named TraN-TraO-TraF in pKM101 and TrwH-TrwF-TrwE in R388) and can be divided into two layers: the O-layer associated with the outer membrane, consisting of VirB7 and the C-terminal domains of VirB9 and VirB10, and the I-layer made up of the N-terminal domains of VirB9 and VirB10 (Figure 3, 4; Fronzes et al., 2009).

The VirB7 lipoprotein component of X-T4SSs is much larger (ranging from 130 to 185 amino acids; Supplementary Figure S7) than that found in other class A T4SSs (normally ∼40 amino acids). This large size is due to an extra globular C-terminal domain called N0 (Souza et al., 2011). Interestingly, similar N0 domains are also found in a myriad of transport systems located in Gram-negative bacterial outer membranes, ranging from secretins of T2SSs, Type IV pilus biogenesis machineries (Korotkov et al., 2009), T3SSs (Spreter et al., 2009), filamentous phages (Spagnuolo et al., 2010), long-tailed bacteriophages (Kanamaru et al., 2002; Kondou et al., 2005), signal-transduction domains in TonB-dependent receptors (Garcia-Herrero and Vogel, 2005; Ferguson et al., 2007) and membrane-penetrating devices in T6SSs (Leiman et al., 2009). The N0 domain is also the C-terminal domain of the outer membrane lipoprotein DotD of the class B T4SSs found in the human pathogens *L. pneumophila* and *C. burnetii* (Nakano et al., 2010). The presence of this domain in many outer membrane transport systems could reflect an unexplored evolutionary relationship between them (Souza et al., 2011). The function of the VirB7 N0 domain is possibly related to the observation that it mediates VirB7 oligomerization and, as the VirB7 subunits are highly concentrated in the context of the core complex, it was predicted that the VirB7 domain could assemble an extra peripheral ring in the O-layer of the X-T4SS (Souza et al., 2011), subsequently confirmed by the resolution of the *X. citri* core complex structure (Sgro et al., 2018). This external ring of N0 domains give the *X. citri* core complex its characteristic profile that resembles a flying saucer (Figure 3; Sgro et al., 2018). The motifs that mediate VirB7 oligomerization (specific residues in the N0 domain and a [T/S]EIPL) motif that immediately precedes it, contribute to T4SS assembly in the *X. citri* periplasm and are essential for its antibacterial activity (Souza et al., 2011; Oliveira et al., 2016; Sgro et al., 2018). The multiple sequence alignment in Supplementary Figure S7 shows that these motifs, as well as the region involved in interaction with VirB9, are conserved among X-T4SS VirB7 proteins.

Like their homologs in other class A T4SSs, the X-T4SS VirB9 proteins have two domains connected by a central linker. They all possess an N-terminal signal peptide with a cleavage site immediately after a highly conserved alanine residue (Supplementary Figure S9). The cryo-EM structure of the *X. citri* core complex (Sgro et al., 2018) and the NMR solution structure of the *X. citri* VirB7–VirB9 binary complex (Oliveira et al., 2016) showed that the *X. citri* VirB9 C-terminal domain interacts with VirB7 and with the VirB10 C-terminal domain in the O-layer of the core complex in a manner similar to that observed for pKM101 (Figure 4). The *X. citri* core complex structure provided us with the first high resolution structure of the I-layer, composed of 14 VirB9 N-terminal β-sandwich domains that pack against each other side-by-side, forming a ring with an internal diameter of 80 Å (Figure 3; Sgro et al., 2018). At the base of the I-layer, small 12-residue helices from the N-terminal domains of VirB10 fit into grooves at the interfaces between VirB9 subunits (Sgro et al., 2018). The multiple sequence alignment of X-T4SS VirB9 proteins shows that the N- and C-terminal domains are quite well conserved but the central linker is variable in both sequence and length (Supplementary Figure S9). This is consistent with the observation that deletion of six residues in the linker region did not affect the stability or function of the T4SS in *X. citri* (Sgro et al., 2018).

VirB10 subunits in class A T4SSs can be divided into three substructures: the N-terminal cytosolic portion with its contiguous transmembrane helix that spans the bacterial inner membrane, the periplasmic portion of the N-terminal domain that is largely unstructured in the NMR analysis of the isolated domain and the cryo-EM structure of the *X. citri* core complex (Sgro et al., 2018) and the C-terminal domain localized in the O-layer of the core complex. The alignments shown in Supplementary Figure S10 reveal that the C-terminal domains of X-T4SS VirB10 subunits are very well conserved. The sequences are also very similar to their counterparts in pKM101, R388 and *A. tumefaciens* (Sgro et al., 2018). One region in this domain that presents a relatively high degree of variability is the “antenna” (Chandran et al., 2009), made up of two alpha helices (α1 and α2) that form the actual pore through the outer membrane. Figure 4 presents a comparison of the relative orientations of the VirB10 antennae when the *X. citri* core complex and pKM101 O-layer structures are superposed. In the *X. citri* core complex structure, the antenna helices are twisted clockwise (∼50°) and tilted vertically (∼20°) with respect to the corresponding helices in pKM101, producing a more open outer membrane pore in the *X. citri* structure (45 Å versus 35 Å; Figure 4; Sgro et al., 2018). We also observe variability in sequence and length of the linker between the alpha helices that is expected to form a loop on the extracellular side of the outer membrane. This loop is particularly rich in threonine, serine and glycine residues and is longer in the Xanthomonadales VirB10 proteins than in their counterparts in *A. tumefaciens* and pKM101 (Supplementary Figure S10; Sgro et al., 2018). In both *X. citri* core complex and pKM101 O-layer structures, these loops are disordered with no significant density in the corresponding maps. Since 14 such loops are expected to be in close proximity at the extracellular face of the outer membrane, they could interact to facilitate closing and opening of the pore. However, an *X. citri* VirB10-msfGFP chimera with an 11 residue deletion in this loop is still partially functional (Sgro et al., 2018).

In contrast to the VirB10 C-terminal domain, its N-terminal domain is known to be highly variable in size and sequence. Xanthomonadales VirB10 proteins have relatively long N-terminal cytosolic segments that precede the transmembrane helix that passes through the inner membrane. While this cytosolic peptide is only 38 or 22 residues long and lacks proline residues in pKM101 and *A. tumefaciens*, it is 57–68 residues long and is rich in proline residues (around 15%) in most X-T4SS VirB10 subunits (Supplementary Figure S10). The N-terminal region of VirB10 has been observed to interact with VirD4 of the T4SSs from *A. tumefaciens* (Garza and Christie, 2013) and plasmids R388 (Llosa et al., 2003; Segura et al., 2013) and R27 (Gilmour et al., 2003). Whether similar interactions occur in the X-T4SS remains to be investigated.

The periplasmic portions of the N-terminal domains (NTDs) of VirB10 subunits in many T4SSs are particularly rich in prolines (Jakubowski et al., 2009) and this is also the case for the X-T4SSs (Supplementary Figure S10; Sgro et al., 2018). NMR studies of the periplasmic VirB10 NTD revealed it to be intrinsically disordered but with a 12 residue segment that samples a helical conformation and suffers conformational perturbations when mixed with a near 2:1 excess of isolated N-terminal domain of VirB9 at sub-millimolar concentrations (Sgro et al., 2018). This VirB10 N-terminal helix (residues 151–162 in *X. citri*; Supplementary Figure S10) fits into the groove between two VirB9 N-terminal domains in the *X. citri* core complex structure (Sgro et al., 2018). The VirB9_NTD_-VirB10_NTD_ interaction, in addition to the VirB7–VirB7 interactions mentioned above (Souza et al., 2011) are two examples of interactions that are relatively weak when measured in isolation but reveal themselves to be physiologically relevant in the context of a large multi-subunit complex whose assembly is expected to be highly cooperative. The helical region is in fact the only well-conserved sequence in the N-terminal domains of the Xanthomonadales and *H. crassostreae* X-T4SS VirB10 subunits and can be described as a P[S/T]Lh[E/D/Q]RRh motif where h is a hydrophobic residue (Supplementary Figure S10). The VirB10 subunits from the two *Neisseria* species shown in Supplementary Figure S10 do not seem to carry this motif.

One interesting observation is the very small number of stereospecific contacts between the I- and O-layers in the *X. citri* core complex. This immediately brings up the question as to what maintains the relative orientations between the two layers. The answer may be that individually weak interactions, multiplied fourteen times in the mature complex, could together be strong enough to favor specific conformational states between the two layers. Another interesting observation is that the long flexible N-terminal linkers that emerge from the VirB9 and VirB10 C-terminal domains in the direction of the I-layer point in opposite directions and pass by each other with their main chain atoms coming within 7 Å of each other (Figure 3C; Sgro et al., 2018). Therefore, the covalent linkages between the VirB9 and VirB10 N- and C-domains can be looked upon as forming an intricate cross-weave pattern at the interface of the I- and O-layers. This detail could have a role in maintaining the two layers in a preferential orientation by restricting the excessive relative rotations in both clockwise and counter-clockwise directions. Enigmatically, however, small (6 or 8 residue) deletions in these linkers had only moderate effects on T4SS-dependent bacterial killing by *X. citri* (Sgro et al., 2018).

### VirB1

In *A. tumefaciens*, VirB1 undergoes cleavage of its N-terminal signal peptide upon transport to the periplasm and a second cleavage reaction that produces two fragments: (i) an N-terminal SLT (soluble lytic transglycosylase) domain predicted to be involved in peptidoglycan remodeling during T4SS biogenesis and (ii) a 76 residue C-terminal fragment (named VirB1^∗^) that is subsequently transported to the extracellular milieu (Baron et al., 1997; Llosa et al., 2000). The VirB1 proteins in the X-T4SSs all have a well-conserved 150 residue N-terminal domain with predicted SLT activity (Supplementary Figure S1). These proteins lack an N-terminal signal sequence, however, and so their mechanism of transport into the periplasm is not yet known. The X-T4SSs VirB1 C-terminal domains vary in length from 130 to over 200 residues and are highly variable in sequence (Supplementary Figure S1). Whether X-T4SS VirB1 proteins undergo C-terminal processing in a manner analogous to that observed in *A. tumefaciens* remains to be investigated.

### VirB2 and VirB5: The Components of the T4SS Pilus

The VirB2 pilin subunits in the Xanthomonadales species and more distant *H. crassostreae*, *N. flavescens*, and *N. mucosa* all have similar sizes and a very well conserved central hydrophobic region as well as a predicted cleavable 31–39 residue long N-terminal signal peptide (Supplementary Figure S2). After removal of the signal peptide, these pilins are predicted to have lengths between 80 and 104 residues. This is significantly greater than the 70 and 64 residue long mature F-pilin and its close homolog from plasmid pED208 (respectively) whose cryo-EM structures have been determined in the context of the assembled sex pilus (Costa et al., 2016). Sequence-based secondary structure predictions for the X-T4SS VirB2 subunits correspond well with the two pilin structures and this allowed us to predict the positions of the two major helices (α2 and α3) in X-T4SS VirB2 as well as the intervening positively charged loop which interacts with the head groups of bound phospholipids in the pilus lumen (Supplementary Figure S2; Costa et al., 2016). The larger size of the X-T4SS VirB2 subunits is due to highly variable C-terminal extensions (Supplementary Figure S2). It is not clear whether these C-terminal extensions decorate the external pilus surface in X-T4SSs, perhaps providing binding sites for species-specific targets, or whether they are processed as has been observed for some P-pili (Eisenbrandt et al., 1999).

VirB5 is thought to be associated with the T4SS pilus, perhaps as a minor pilin at the pilus tip (Schmidt-Eisenlohr et al., 1999; Aly and Baron, 2007; Alvarez-Martinez and Christie, 2009). Due to the very high sequence variability in VirB5 proteins, its annotation as a bona-fide T4SS component in deposited genomic sequences is often ambiguous. In the bacteria-killing T4SSs under consideration here, these proteins have between 200 and 280 residues and are highly variable in sequence. They all have a predicted N-terminal cleavable signal peptide as well as a pair of cysteine residues found in the central portion of the amino acid sequence that are separated by 11 to 33 residues (Supplementary Figure S5). Interestingly, the genetic loci coding for X-T4SSs in the two *Lysobacter* species shown in Figure 2 each carry two *virB5* genes in tandem. These protein pairs are 73% and 43% identical in *Lysobacter antibioticus* and *Lysobacter enzymogenes*, respectively. The predicted involvement of VirB5 in mediating the binding of the pilus to specific structures on the target cell (Alvarez-Martinez and Christie, 2009), could be a causative factor in this subunit’s accelerated evolution.

### VirB8, VirB6, and VirB3: Integral Membrane Proteins of the Inner Membrane Complex

VirB8 is an integral membrane protein with an N-terminal cytosolic peptide, a transmembrane helix and a globular C-terminal domain localized in the periplasm, the latter of which has been shown to interact with several other T4SS components, including VirB6, VirB9, and VirB10 (Alvarez-Martinez and Christie, 2009; Sivanesan et al., 2010; Villamil Giraldo et al., 2012). High resolution structures of the soluble C-terminal region of VirB8 proteins and homologs from diverse T4SSs have been determined: *A. tumefaciens* (Bailey et al., 2006), pKM101 (Casu et al., 2016), *H. pylori* (ComB10) and *Brucella suis* (Terradot et al., 2005), *Clostridium perfringens*, *Rickettsia typhi* and several *Bartonella* species (Gillespie et al., 2015), *L. pneumophila* and plasmid R64 (Kuroda et al., 2015). All these structures present the same fold, a β-sheet juxtaposed against a group of α-helices, and in most cases have been shown to oligomerize to different degrees (see references above). Since there are an estimated 12 copies of VirB8 in each class A T4SS (Low et al., 2014), these domains could associate to form an as-yet unknown structure in the bacterial periplasm. Interestingly, the bacteria-killing X-T4SSs have highly distinctive VirB8 components that are significantly longer (between 290 and 370 residues in length) than the canonical VirB8 components observed in the species listed above (all less than 250 residues). This greater size is, in large part, due to a C-terminal extension enriched in Ala, Gln, Gly, and Pro (AQGP) residues (Table 2 and Supplementary Figure S8). The length of this extension and its enrichment in these residues are particularly evident in the Xanthomonadales order (53–73%) and *H. crassostreae* (45%) and less so in the X-T4SS VirB8 proteins of *Neisseria* species (Table 2). The role, if any, of the AQGP-rich extensions in these proteins is not yet known.

**Table 2:** Characteristics of C-terminal extensions in VirB8 proteins from X-T4SSs.

Organism	Extension size (aa)	% Ala	% Gln	% Gly	% Pro	% AQGP
*Xanthomonas citri* 306	98	21	25	11	15	72
*Stenotrophomonas maltophilia* K279a	104	19	19	12	15	65
*Lysobacter antibioticus* 76	97	26	4	10	32	72
*Lysobacter enzymogenes* C3	100	25	7	10	31	73
*Luteibacter rhizovicinus* DSM16549	129	26	11	12	22	71
*Dyella jiangningensis* SBZ3-12	70	16	9	11	17	53
*Dyella thiooxydans* ATSB10	48	33	2	8	27	70
*Hydrogenophaga crassostreae* LPB0072	53	13	6	11	15	45
*Neisseria mucosa* C102	55	6	16	4	13	39
*Neisseria flavescens* SK114	52	6	14	6	6	32

*Analysis is based on the alignment shown in Supplementary Figure S8.*

VirB6 is predicted to be a polytopic integral protein. In both *A. tumefaciens* and *B. suis*, the VirB6 N-terminus is located in the periplasm and the C-terminus is located in the cytosol, implying an odd number of transmembrane helices, estimated to be five in *A. tumefaciens* (Jakubowski et al., 2004) and seven in *B. suis* (Villamil Giraldo et al., 2015). Supplementary Figure S6 presents the multiple sequence alignment of X-T4SS VirB6 proteins that also align well with their homologs in *A. tumefaciens* and *B. suis* (data not shown). The precise number and positions of the transmembrane helices is again ambiguous since transmembrane helix pairs 3/4 and 5/6 could alternatively be longer single helices (Supplementary Figure S6). Another common feature of these proteins is a large loop between transmembrane helices 2 and 3, predicted to be localized in the periplasm for both *A. tumefaciens* and *B. suis*. The periplasmic loops of the estimated 24 copies of VirB6 per T4SS (Low et al., 2014) are expected to interact with other periplasmic components of the inner membrane complex (Figure 1; Ding et al., 2003; Alvarez-Martinez and Christie, 2009; Villamil Giraldo et al., 2012, 2015; Christie, 2016).

X-T4SS VirB3 proteins are predicted to be bitopic or polytopic membrane proteins with one or two transmembrane helices located within the region encompassed by residues 15 and 57 (see Supplementary Figure S3 for details). This is consistent with the difficulty in defining VirB3 topology in other T4SSs (Alvarez-Martinez and Christie, 2009). In some organisms, gene fusions have been observed between VirB3 and VirB4 (Batchelor et al., 2004) but this does not seem to be the case in the X-T4SSs. These gene fusions and the highly conserved synteny of *virB3* and *virB4* genes in most organisms suggests that these two proteins could interact with each other at or within the inner membrane (Peña et al., 2012). This hypothesis is supported by the observation that *A. tumefaciens* VirB4 is required to maintain normal levels of VirB3 (Jones et al., 1994) and that recombinant R388 VirB4 and VirB3 can be co-purified as a 1:1 complex (Low et al., 2014).

### VirB4, VirB11, and VirD4: The ATPases of the Inner Membrane Complex

VirB4, VirB11, and VirD4 are P-loop ATPases with conserved Walker A and Walker B motifs and, in most T4SSs characterized to date, all three proteins are required for biogenesis and/or function (Alvarez-Martinez and Christie, 2009; Christie et al., 2014; Christie, 2016). These proteins use the hydrolysis of ATP to carry out mechanical work, expected to manifest itself in substrate unfolding, transfer and/or extrusion through the T4SS channel (Atmakuri et al., 2004).

VirB4 is the most well conserved T4SS subunit and has been used in phylogenetic analyses to trace evolutionary relationships and propose models for the emergence of T4SS subclasses (Guglielmini et al., 2014). Studies on different T4SSs have reported evidence that VirB4 interacts, at least transiently, with all of the other inner membrane complex components (Jones et al., 1994; Atmakuri et al., 2004; Cascales and Christie, 2004; Jakubowski et al., 2004; Paschos et al., 2006; Ripoll-Rozada et al., 2013; Low et al., 2014; Christie, 2016).

VirB4 has been localized to two 3-tiered barrel-like pedestals at the base of the inner membrane complex in EM reconstructions of the complete R388 T4SS, with each barrel corresponding to a VirB4 hexamer (Low et al., 2014; Redzej et al., 2017). VirB4 proteins can be divided into N- and C-terminal domains. The C-terminal domain carries all the conserved motifs required for nucleotide binding and hydrolysis (Walker A and Walker B boxes and motifs C, D, and E) and these motifs are present in X-T4SS VirB4 proteins (Supplementary Figure S4). The N-terminal domain, known to mediate interactions with the inner membrane, is expected to correspond to the upper tier in the R388 T4SS structure (Low et al., 2014; Redzej et al., 2017). One variable in the family of VirB4 proteins is the presence or absence of one or more predicted N-terminal transmembrane helices and the question as to their requirement for VirB4 function has proven to be controversial (Rabel et al., 2003), with the additional caveat that VirB4/TraB from pKM101 can be purified in soluble and membrane-bound forms (Durand et al., 2010). Therefore, it is not clear whether VirB4 should be considered an integral or peripheral membrane protein, or both (Arechaga et al., 2008; Christie, 2016; Waksman, 2019). The X-T4SS VirB4 proteins do not have predicted transmembrane helices using the TMHMM v2.0 (Krogh et al., 2001) and PSort (Nakai and Horton, 1999) prediction algorithms.

VirB11 is a soluble membrane-associated AAA+ ATPase that has been shown to interact with both VirD4 and VirB4 (Ripoll-Rozada et al., 2013). The crystal structures of VirB11 homologs from *B. suis* (Hare et al., 2006) and *H. pylori* (Yeo et al., 2000) are both two-tiered hexameric rings in which each ring layer consists of six N- or C-terminal domains from the constituent monomers. Within VirB11 monomers, the domains are connected by a central linker of varying length, short in *H. pylori* and 17 residues longer (linker B and α2C) in *B. suis*, resulting in a domain-swapped architecture in the latter (Hare et al., 2006). As a consequence, the nucleotide binding site in *H. pylori* VirB11 is located at the interface between the two domains of the same monomer while in *B. suis* the nucleotide binds at the interface between the N-terminal domain of one monomer and the C-terminal domain of the neighboring monomer (Hare et al., 2006). VirB11 proteins from X-T4SSs show a high degree of sequence similarity and all have the long version of the linker, which aligns well with the *B. suis* linker B and α2C sequences (Supplementary Figure S11). We can therefore expect that the X-T4SS VirB11 proteins exhibit a domain-swapped structure similar to that of *B. suis* (Hare et al., 2006).

The VirD4 ATPase and its homologs are often called coupling proteins due to their role in selecting substrates for export by the T4SS. Analysis of the X-T4SS VirD4 proteins suggests that they have a canonical VirD4-like architecture (Llosa and Alkorta, 2017) with two N-terminal transmembrane helices with a predicted intervening ∼30 amino acid periplasmic loop and a cytosolic C-terminal domain. The cytosolic domain can be separated into a nucleotide binding domain (NBD), with conserved Walker A and Walker B motifs, and a so-called all alpha domain (AAD) (Supplementary Figure S12; Gomis-Ruth et al., 2002). The VirD4 N-terminal transmembrane domain helices have been implicated in interacting with the VirB10 N-terminal region that includes its transmembrane helix (Segura et al., 2013) and the VirD4 all alpha domain is involved in substrate recognition (Gomis-Ruth et al., 2002; Whitaker et al., 2015). TrwB, the VirD4 homolog of the conjugative plasmid R388, has been crystallized as a hexameric ring (Gomis-Ruth et al., 2002). It has been proposed that VirB4 and VirD4 could form transient heterohexameric complexes during substrate transport (Peña et al., 2012; Waksman, 2019) and low resolution electron microscopy studies of the intact VirB3-B10/D4 T4SS from R388 observed VirD4 dimers sandwiched between the two hexameric VirB4 barrels (Redzej et al., 2017). Therefore, the oligomeric structure of VirD4 in a fully assembled and functioning T4SS is still not clear (Redzej et al., 2017; Chetrit et al., 2018; Waksman, 2019).

## Xanthomonadales-Like T4SS Effectors (X-Tfes) and Their Cognate Inhibitors (X-Tfis)

### X-Tfes

**FIGURE 5:**
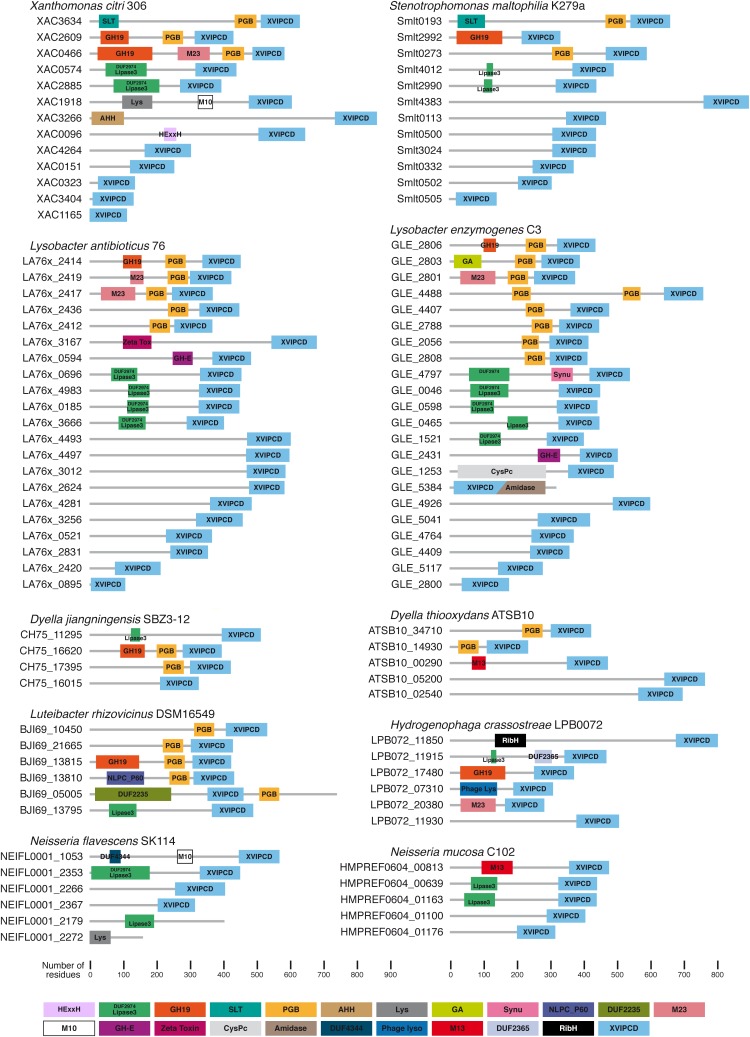
Putative Xanthomonadales-like T4SS effectors (X-Tfes) found in selected species that carry an X-T4SS. Shown here are representative examples from *Xanthomonas citri* 306 (Da Silva et al., 2002), *Stenotrophomonas maltophilia* K279a (Crossman et al., 2008), *Lysobacter antibioticus* 76 (de Bruijn et al., 2015), *Lysobacter enzymogenes* C3 (unpublished; GenBank accession CP013140), *Luteibacter rhizovicinus* DSM16549 (unpublished; GenBank accession CP017480), *Dyella jiangningensis* SBZ3-12 (Bao et al., 2014), *Dyella thiooxydans* strain ATSB10 (unpublished; GenBank accession CP014841), *Hydrogenophaga crassostreae* LPB0072 (unpublished; GenBank accession LVWD01000013), *Neisseria mucosa* C102 (unpublished, GenBank accession GCA_000186165) and *Neisseria flavescens* SK114 (unpublished; GenBank accession ACQV01000009). Protein domains were identified by sequence comparison with the Pfam (El-Gebali et al., 2019) and/or CDD databases (Marchler-Bauer et al., 2015) and are colored according to the scheme presented at the bottom of the Figure. Domain abbreviations: M10 (Pfam accession PF08548), M13 (Pfam accession PF01431), M23 (Pfam accession PF01551), Lipase3 (Pfam accession PF01764), DUF2974 (Pfam accession PF11187), GH-E (Pfam accession PF14410), GH19 (Pfam accession PF00182), Zeta Toxin (Pfam accession PF06414), SLT (CDD accession cd00254), CysPc (CDD accession cd00044), PGB (Pfam accession PF01471), Amidase (Pfam accession PF01510), AHH (Pfam accession PF14412), DUF4344 (Pfam accession PF14247), Lys (CDD accession cl00222), Phage lyso (Pfam accession PF00959), GA (Pfam accession PF01832), Synu (CDD accession cl03193), DUF2365 (Pfam accession PF10157), NLPC_P60 (Pfam accession PF00877), RibH (Pfam accession PF02267), DUF2235 (Pfam accession PF09994), HExxH (HExxH motif in putative metalloprotease domain; Firczuk and Bochtler, 2007).

The first clues regarding the physiological role of the *X. citri* T4SS came from the identification of T4SS substrates using the VirD4 coupling protein as a bait in yeast two-hybrid assays against a *Xanthomonas* genomic library (Alegria et al., 2005). The strategy was based on the reasoning that in other well-characterized T4SSs, the VirD4 component is known to interact with the macromolecular substrates prior to transport (Llosa and Alkorta, 2017). This screening originally identified 12 so-called “*Xanthomonas* VirD4 interacting proteins,” or XVIPs (Alegria et al., 2005), later called *Xanthomonadaceae* T4SS effectors (Souza et al., 2015) and from here on Xanthomonadales-like T4SS effectors (X-Tfes, Figure 5). In *X. citri*, the gene for one X-Tfe (XAC2609) is found in the *vir* locus while the remaining X-Tfe genes are dispersed throughout the genome. Interestingly, all of these proteins have a common C-terminal domain entitled “XVIP conserved domain” or XVIPCD (Figure 1, 5), typically around 120 residues long, required for interaction with VirD4 (Alegria et al., 2005) and for secretion in a T4SS-dependent manner (Souza et al., 2015). The XVIPCD is characterized by a few conserved motifs in its N-terminal region and a glutamine-rich C-terminal region (Alegria et al., 2005).

The discovery of the XVIPCD as the secretion signal for the *X. citri* X-Tfes allowed for a large-scale bioinformatics identification of X-Tfe genes present in other bacterial genomes (Souza et al., 2015). Figure 5 shows the domain architectures of the X-Tfes identified by bioinformatics analysis of bacterial genomes whose X-T4SSs are described in Figure 2. The N-terminal portions of the *X. citri* X-Tfes are highly variable in size and architecture and most are predicted to function within the periplasm as peptidoglycan (PG) glycohydrolases, lytic transglycosylases, PG peptidases or lipases (Figure 5). Therefore, these bacterial species probably use their X-T4SSs to inject not one, but a diverse cocktail of X-Tfes that will simultaneously attack multiple structures in the target cell (Figure 1). Two purified *X. citri* X-Tfes (XAC2609 and XAC0466) with predicted PG hydrolase activities have been shown to lyse PG and induce the lysis of Gram-positive cells, which have exposed bacterial cell walls (Souza et al., 2015). It is interesting that a considerable fraction of X-Tfes have N-terminal sequences with no identifiable domains, opening the possibility that new domain families with antibacterial activities could be characterized in the future. One such X-Tfe, Smlt3024 from *S. maltophilia* K279a (Figure 5), has been shown to inhibit *E. coli* growth when heterologously expressed and directed to the periplasm (preprint: Bayer-Santos et al., 2019).

It is worth noting that we often encounter several open reading frames that code for small proteins, sometimes possessing little more than an intact XVIPCD; for example XAC0323, XAC1165, and XAC3404 in *X. citri* (respectively 136, 127, and 132 residues in length; Figure 5; Souza et al., 2015). In some cases, these open reading frames appear to be fragments of ancestral X-Tfes genes that suffered frameshift mutations. One example of this phenomenon is provided by the XAC1165 gene whose first 37 nucleotides overlap with the 3′ end of the upstream XAC1164 gene which codes for a 437 protein of unknown function. The amino acid sequences of XAC1164 and XAC1165 align very well with the N-terminal and XVIPCD regions, respectively, of the Smlt0113 X-Tfe protein from *S. maltophilia* (Figure 5). Thus *X. citri* XAC1164 and XAC1165 proteins are homologous, and probably the non-functional, fragments of a functional X-Tfe (Smlt0113) in *S. maltophilia*.

### X-Tfis

**FIGURE 6:**
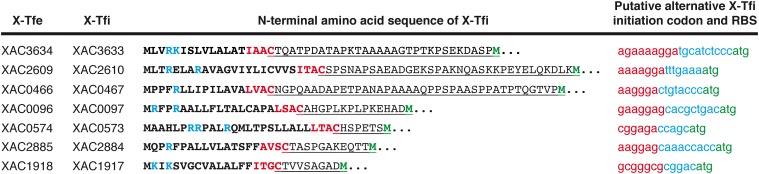
Possible alternative translation start codons that could lead to the production of soluble cytosolic X-Tfis in *Xanthomonas citri*. The first two columns list the names of *X. citri* X-Tfe/X-Tfi pairs in which the X-Tfi is predicted to be a lipoprotein (Souza et al., 2015). The third column presents the N-terminal amino acid sequence of the X-Tfi in which the signal sequence and Lipobox are shown in bold. The basic nucleotides at the N-terminus of the signal sequence are shown in blue. The four Lipobox residues are shown in red. Underlined residues are those from the absolutely conserved Cys residue at the site of cleavage in the Lipobox to the next Met residue (green) in the protein sequence. The last column presents the nucleotide sequence (lowercase letters) immediately upstream of the putative alternative start codon (green). The putative Shine–Dalgarno sequence (ribosome binding site) for this alternative start codon is shown in red.

To protect against the toxicity of endogenous or exogenous X-Tfes, *X. citri* and *S. maltophilia* produce specific immunity proteins that bind to their cognate toxins (Alegria et al., 2005; Souza et al., 2015; preprint: Bayer-Santos et al., 2019). These inhibitors have been termed *Xanthomonadaceae* T4SS immunity proteins (Souza et al., 2015) and from here on Xanthomonadales-like T4SS immunity proteins (X-Tfis). The genes coding for X-Tfis are usually found upstream and are probably co-transcribed with their cognate X-Tfe (Souza et al., 2015; preprint: Bayer-Santos et al., 2019). All bacterial species identified so far that carry an X-T4SS also code for multiple X-Tfe/X-Tfi pairs and, in most cases, the genes for at least one pair is found within, or in close proximity to, the locus that codes for the structural components of the X-T4SS (see Figure 2). Furthermore, in almost all cases where the X-Tfe is predicted to act upon periplasmic structures (glycosidic and peptide bonds in peptidoglycan or ester linkages in phospholipids), the cognate X-Tfi carries an N-terminal signal peptide and lipobox for periplasmic localization and anchoring in the outer membrane (Souza et al., 2015; preprint: Bayer-Santos et al., 2019). On the other hand, some X-Tfes with N-terminal domains predicted to act in the cytosol of the target cell (for example the *X. citri* X-Tfe XAC3266 with an N-terminal AHH domain with predicted nuclease activity; Figure 5) have a cognate X-Tfi (for example XAC3267) lacking a lipoprotein signal (Souza et al., 2015). Finally, some X-Tfes are expected to be active in both the cytosol and periplasm, as are the cases of the X-Tfes with predicted lipase domains with phospholipase activities (NFB, DPS, BM, and CSF, manuscript in preparation; Figure 5). This brings up the question regarding the cellular localization of the X-Tfis. An analysis of the *X. citri* X-Tfis with putative N-terminal signal peptide and Lipobox sites indicates that their coding genes have potential alternative downstream start (ATG) codons with associated ribosome binding sites (Figure 6). This raises the possibility that many X-Tfes can be produced in two versions: (i) a membrane-associated periplasmic lipoprotein and (ii) a soluble cytosolic protein (Figure 1). Thus, if X-Tfes make their way into the periplasm, either by leakage from the secretion channel or by injection by neighboring cells of the same species, they will be inhibited by the periplasmic lipoprotein forms of the cognate X-Tfi. On the other hand, X-Tfes whose activities could target cytosolic substrates can be inhibited by cytosolic variants of their cognate X-Tfis. If this is in fact the case, for at least this latter subset of X-Tfes, transport will necessarily involve previous dissociation of the X-Tfe/X-Tfi cytosolic pair.

### Parallels Between X-T4SS X-Tfe/X-Tfi, T6SS Effector/Immunity Protein and Plasmid-Encoded Toxin/Antitoxin (TA) Pairs

X-Tfe/X-Tfi pairs share many of the characteristics observed for T6SS effectors and their inhibitors (Russell et al., 2011, 2013). For example, one immunity protein from *X. citri* (X-Tfi^XAC2610^) inhibits the GH19 family PG hydrolase X-Tfe^XAC2609^ and has a very similar topology, though very little sequence similarity, to the PG hydrolase inhibitors PliI and Tsi1 (Van Herreweghe et al., 2010; Russell et al., 2011) the latter of which is an inhibitor of the T6SS effector Tse1 from *Pseudomonas aeruginosa* (Souza et al., 2015). Another example is provided by the X-Tfe Smlt0332 from *S. maltophilia* K279a, which has many X-Tfe homologs in other Xanthomonadales species (data not shown) but whose N-terminal region has no similarity with annotated domains in the Pfam or CCD databases. Interestingly, Blast searches against the curated KEGG database (Kanehisa et al., 2017) using this domain identified a large number of homologous sequences fused to VgrG domains in effectors predicted to be secreted by T6SSs of *Metakosakonia*, *Pantoea*, *Kosakonia*, *Enterobacter*, *Burkholderia*, *Methylocaldum*, *Ralstonia*, *Cronobacter*, and *Xanthomonas* species (Table 3). Searches against the non-redundant protein sequence database (Altschul et al., 1990) identified many more such homologs in other bacterial species (data not shown). These findings raise interesting questions regarding the evolution, distribution and exchange of T4SS and T6SS effectors in the biosphere. In fact, we can make a general observation that anti-bacterial Type IV and Type VI secretion systems share many enzymatic effector and cognate inhibitory modules and differ only in the specific sequences recognized for transport. It also raises the possibility that the acquisition of immunity proteins could be advantageous even in the absence of the cognate effector by offering a defense against the toxic activity of substrates launched by both T4SS and T6SSs during encounters with rival bacteria. We have in fact recently observed the reciprocal T4SS-dependent dueling between *S. maltophilia* and *X. citri* cells which could mimic similar encounters between soil- and/or plant-associated bacteria in the environment (preprint: Bayer-Santos et al., 2019). The differences in X-Tfe and X-Tfi repertoires between rival species will contribute to the outcome of these encounters.

**Table 3:** List of proteins in the KEGG database with greatest similarity^a^ to the N-terminal domain (residues 1–240) of Smlt0332 from *S. maltophilia* K279a.

Organism	Accession^b^	*E*-value	Description
*Xanthomonas campestris* pv. *campestris* B100	xcc-b100_0624	3 E-88	X-T4SS X-Tfe
*Xanthomonas campestris* pv. *campestris* 8004	XC_3909	2 E-83	X-T4SS X-Tfe
*Xanthomonas campestris* pv. *campestris* ATCC33913	XCC3567	2 E-83	X-T4SS X-Tfe
*Xanthomonas vasicola* pv. *vasculorum* SAM119	C7V42_12340	2 E-79	X-T4SS X-Tfe
*Xanthomonas vesicatoria* ATCC35937	BJD12_15640	2 E-76	X-T4SS X-Tfe
*Metakosakonia* sp. MRY16-398	MRY16398_33930	2 E-26	Type VI secretion system secreted protein VgrG
*Pantoea ananatis* LMG20103	PANA_2352	9 E-26	Type VI secretion system secreted protein VgrG
*Pantoea vagans* FDAARGOS_160	AL522_12495	4 E-25	Type VI secretion system secreted protein VgrG
*Kosakonia radicincitans* GXGL-4A	A3780_13240	4 E-22	Type VI secretion system secreted protein VgrG/Rhs
*Enterobacter hormaechei* subsp. *xiangfangensis* LMG27195	BFV63_12735	4 E-21	Type VI secretion system secreted protein VgrG/Rhs
*Enterobacter hormaechei* subsp. *xiangfangensis* 34399	LI66_12735	9 E-21	Type VI secretion system secreted protein VgrG/Rhs
*Enterobacter hormaechei* subsp. *hoffmannii* ECR091	ECR091_12370	9 E-21	Type VI secretion system secreted protein VgrG
*Enterobacter hormaechei* subsp. *hoffmannii* ECNIH3	ECNIH3_12435	2 E-20	Type VI secretion system secreted protein VgrG
*Enterobacter cloacae* ECNIH5	ECNIH5_12380	3 E-20	Type VI secretion system secreted protein VgrG/Rhs
*Burkholderia stabilis* ATCCBAA-67	BBJ41_25130	2 E-20	Type VI secretion system secreted protein VgrG
*Enterobacter hormaechei* subsp. *hormaechei* 34983	LI64_12500	2 E-20	Type VI secretion system secreted protein VgrG/Rhs
*Methylocaldum marinum* S8	sS8_3556	6 E-20	Type VI secretion system secreted protein VgrG
*Ralstonia solanacearum* FQY_4	F504_2863	2 E-19	Type VI secretion system secreted protein VgrG
*Ralstonia solanacearum* Po82	RSPO_c00015	2 E-19	Type VI secretion system secreted protein VgrG
*Burkholderia territorii* RF8-non-BP5	WS51_08400	4 E-19	Type VI secretion system secreted protein VgrG
*Cronobacter turicensis* z3032	CTU_00910	3 E-19	Type VI secretion system secreted protein VgrG
*Ralstonia pseudosolanacearum* RS 476	CDC45_17535	5 E-19	Type VI secretion system secreted protein VgrG
*Ralstonia solanacearum* GMI1000	RSc3430	5 E-19	Type VI secretion system secreted protein VgrG
*Ralstonia solanacearum* PSI07	RPSI07_0016	1 E-17	Type VI secretion system secreted protein VgrG
*Burkholderia stagnalis* MSMB735WGS	WT74_22275	3 E-17	Type VI secretion system secreted protein VgrG
*Xanthomonas fragariae* Fap21	BER92_04245	2 E-17	Type VI secretion system secreted protein VgrG
*Ralstonia solanacearum* FQY_4	F504_3476	4 E-17	Type VI secretion system secreted protein VgrG
*Xanthomonas campestris* pv. *raphani* 756C	XCR_2915	4 E-17	Hypothetical protein
*Mycolicibacterium hassiacum* DSM44199	MHAS_03665	1 E-16	Hypothetical protein
*Burkholderia ubonensis* MSMB22	BW23_4367	3 E-16	Type VI secretion system secreted protein VgrG/Rhs
*Enterobacter* sp. R4-368	H650_00935	2 E-12	Type VI secretion system secreted protein VgrG
*Kosakonia cowanii* 888-76	BWI95_18245	7 E-09	Type VI secretion system secreted protein VgrG/Rhs

*^a^only proteins with significant coverage in the alignment are shown. ^b^KEGG (Kyoto Encyclopedia of Genes and Genomes) accession number.*

Effector/immunity protein pairs associated with T4SS and T6SSs show intriguing parallels with toxin/anti-toxin modules that function to guarantee vertical transmission of mobile elements (Jensen and Gerdes, 1995). For example, Harms et al. (2017) have shown that the pVbh plasmid of *Bartonella schoenbuchensis*, codes for a toxin/antitoxin module in which the toxin component, VbhT, acquired a C-terminal BID (Bep Intracellular Delivery) domain that confers its transfer to recipient cells during conjugation. They propose that its function may be to support intercellular DNA transfer by pre-emptively addicting the recipient cell to the plasmid, which also carries the gene for the antitoxin antidote. It is not difficult to imagine a scenario in which the T4SS coded by such a plasmid could lose its capacity to transfer DNA but retain its capacity to transfer the toxin, and in this way evolve into a single-purpose bacteria-killing T4SS. Therefore, we can expect bactericidal T4SSs, perhaps with very different recognition signals, to have arisen on multiple occasions in distantly related bacterial species.

## Closing Remarks

These are early days in the characterization of antibacterial T4SSs. The structure of the *X. citri* core complex has provided a good reference for comparison with other T4SSs, and has illustrated the structural variability that we can expect to encounter even within the class A T4SSs. While X-T4SS activities have only been experimentally verified for *X. citri* and *S. maltophilia*, bioinformatics analysis allowed us to confidently expand the list of bacterial families both within the Gammaproteobacteria class as well as to other families within the Betaproteobacteria (Table 1) that carry proteins with many of the characteristic X-T4SSs features, including VirB7 proteins with N0 domains, VirB8 proteins with AQGP-rich extensions as well as recognizable X-Tfe/X-Tfi pairs in which the effector carries a C-terminal XVIPCD. The list of X-T4SSs will most surely expand significantly in the future. However, it is unlikely that bactericidal T4SSs are restricted to the X-T4SSs described here. It is more probable that many other bacterial species carry as yet uncharacterized and perhaps unrecognized T4SSs that recruit effectors with recognition signals significantly different from the XVIPCDs associated with X-T4SSs as predicted (Souza et al., 2015) and as illustrated by the results obtained for the T4SS encoded by the *B. schoenbuchensis* pVbh plasmid (Harms et al., 2017).

## Author Contributions

GS, GO, EB-S, and CF produced the figures and tables. CF wrote the manuscript. All authors contributed with critical discussions and revisions that led to the final version of the manuscript.

## Conflict of Interest Statement

The authors declare that the research was conducted in the absence of any commercial or financial relationships that could be construed as a potential conflict of interest.

## References

[B1] Alegria M. C. Souza D. P. Andrade M. O. Docena C. Khater L. Ramos C. H. I. (2005). Identification of new protein-protein interactions involving the products of the chromosome- and plasmid-encoded type IV secretion loci of the phytopathogen *Xanthomonas axonopodis* pv. *citri*. *J. Bacteriol.* 187 2315–2325. 10.1128/JB.187.7.2315-2325.2005 15774874 PMC1065226

[B2] Altschul S. F. Gish W. Miller W. Myers E. W. Lipman D. J. (1990). Basic local alignment search tool. *J. Mol. Biol.* 215 403–410. 10.1016/S0022-2836(05)80360-2 2231712

[B3] Alvarez-Martinez C. E. Christie P. J. (2009). Biological diversity of prokaryotic type IV secretion systems. *Microbiol. Mol. Biol. Rev.* 73 775–808. 10.1128/MMBR.00023-09 19946141 PMC2786583

[B4] Aly K. A. Baron C. (2007). The VirB5 protein localizes to the T-pilus tips in *Agrobacterium tumefaciens*. *Microbiology* 153 3766–3775. 10.1099/mic.0.2007/010462-0 17975085

[B5] Arechaga I. Pena A. Zunzunegui S. del Carmen Fernandez-Alonso M. Rivas G. de la Cruz F. (2008). ATPase activity and oligomeric state of TrwK, the VirB4 homologue of the plasmid R388 type IV secretion system. *J. Bacteriol.* 190 5472–5479. 10.1128/JB.00321-08 18539740 PMC2493248

[B6] Atmakuri K. Cascales E. Christie P. J. (2004). Energetic components VirD4, VirB11 and VirB4 mediate early DNA transfer reactions required for bacterial type IV secretion. *Mol. Microbiol.* 54 1199–1211. 10.1111/j.1365-2958.2004.04345.x 15554962 PMC3869561

[B7] Backert S. Meyer T. F. (2006). Type IV secretion systems and their effectors in bacterial pathogenesis. *Curr. Opin. Microbiol.* 9 207–217. 10.1016/j.mib.2006.02.008 16529981

[B8] Backert S. Tegtmeyer N. Fischer W. (2015). Composition, structure and function of the *Helicobacter pylori* cag pathogenicity island encoded type IV secretion system. *Future Microbiol.* 10 955–965. 10.2217/fmb.15.32 26059619 PMC4493163

[B9] Bailey S. Ward D. Middleton R. Grossmann J. G. Zambryski P. C. (2006). *Agrobacterium tumefaciens* VirB8 structure reveals potential protein-protein interaction sites. *Proc. Natl. Acad. Sci. U.S.A.* 103 2582–2587. 10.1073/pnas.0511216103 16481621 PMC1413848

[B10] Bao Y. Kwok A. H. Y. He L. Jiang J. Huang Z. Leung F. C. C. (2014). Complete genome sequence of *Dyella jiangningensis* strain SBZ3-12, isolated from the surfaces of weathered rock. *Genome Announc.* 2:e00416-14. 10.1128/genomeA.00416-14 24831147 PMC4022811

[B11] Baron C. Llosa M. Zhou S. Zambryski P. C. (1997). VirB1, a component of the T-complex transfer machinery of *Agrobacterium tumefaciens*, is processed to a C-terminal secreted product, VirB1. *J. Bacteriol.* 179 1203–1210. 10.1128/jb.179.4.1203-1210.1997 9023203 PMC178817

[B12] Batchelor R. A. Pearson B. M. Friis L. M. Guerry P. Wells J. M. (2004). Nucleotide sequences and comparison of two large conjugative plasmids from different *Campylobacter* species. *Microbiology* 150(Pt 10), 3507–3517. 10.1099/mic.0.27112-0 15470128

[B13] Bayer-Santos W. Matsuyama B. Y. Di Sessa G. Mininel I. D. V. Farah C. S. (2019). The opportunistic pathogen *Stenotrophomonas maltophilia* utilizes a type IV secretion system for interbacterial killing. *bioRxiv* [Preprint]. 10.1101/557322 PMC6759196 31513674

[B14] Bayer-Santos E. Lima L. dos P. Ceseti L. de M. Ratagami C. Y. (2018). *Xanthomonas citri* T6SS mediates resistance to *Dictyostelium* predation and is regulated by an ECF σ factor and cognate Ser/Thr kinase. *Environ. Microbiol.* 20 1562–1575. 10.1111/1462-2920.14085 29488354

[B15] Brito E. M. Pinon-Castillo H. A. Guyoneaud R. Caretta C. A. Gutierrez-Corona J. F. Duran R. (2013). Bacterial biodiversity from anthropogenic extreme environments: a hyper-alkaline and hyper-saline industrial residue contaminated by chromium and iron. *Appl. Microbiol. Biotechnol.* 97 369–378. 10.1007/s00253-012-3923-5 22350256

[B16] Buttner D. Bonas U. (2010). Regulation and secretion of *Xanthomonas* virulence factors. *FEMS Microbiol. Rev.* 34 107–133. 10.1111/j.1574-6976.2009.00192.x 19925633

[B17] Cabezon E. Ripoll-Rozada J. Pena A. de la Cruz F. Arechaga I. (2015). Towards an integrated model of bacterial conjugation. *FEMS Microbiol. Rev.* 39 81–95. 10.1111/1574-6976.12085 25154632

[B18] Callaghan M. M. Heilers J. H. van der Does C. Dillard J. P. (2017). Secretion of chromosomal DNA by the *Neisseria gonorrhoeae* type IV secretion system. *Curr. Top. Microbiol. Immunol.* 413 323–345. 10.1007/978-3-319-75241-9_13 29536365 PMC5935271

[B19] Cao Z. Casabona M. G. Kneuper H. Chalmers J. D. Palmer T. (2016). The type VII secretion system of *Staphylococcus aureus* secretes a nuclease toxin that targets competitor bacteria. *Nat. Microbiol.* 2:16183. 10.1038/nmicrobiol.2016.183 27723728 PMC5325307

[B20] Carbonetti N. H. (2015). Contribution of pertussis toxin to the pathogenesis of pertussis disease. *Pathog. Dis.* 73:ftv073. 10.1093/femspd/ftv073 26394801 PMC4626579

[B21] Cascales E. Christie P. J. (2004). Definition of a bacterial type IV secretion pathway for a DNA substrate. *Science* 304 1170–1173. 10.1126/science.1095211 15155952 PMC3882297

[B22] Casu B. Smart J. Hancock M. A. Smith M. Sygusch J. Baron C. (2016). Structural analysis and inhibition of TraE from the pKM101 type IV secretion system. *J. Biol. Chem.* 291 23817–23829. 10.1074/jbc.M116.753327 27634044 PMC5095433

[B23] Chandran V. Fronzes R. Duquerroy S. Cronin N. Navaza J. Waksman G. (2009). Structure of the outer membrane complex of a type IV secretion system. *Nature* 462 1011–1015. 10.1038/nature08588 19946264 PMC2797999

[B24] Chang Y. T. Lin C. Y. Chen Y. H. Hsueh P. R. (2015). Update on infections caused by *Stenotrophomonas maltophilia* with particular attention to resistance mechanisms and therapeutic options. *Front. Microbiol.* 6:893. 10.3389/fmicb.2015.00893 26388847 PMC4557615

[B25] Chang Y. W. Shaffer C. L. Rettberg L. A. Ghosal D. Jensen G. J. (2018). In vivo structures of the *Helicobacter pylori* cag type IV secretion system. *Cell Rep.* 23 673–681. 10.1016/j.celrep.2018.03.085 29669273 PMC5931392

[B26] Chetrit D. Hu B. Christie P. J. Roy C. R. Liu J. (2018). A unique cytoplasmic ATPase complex defines the *Legionella pneumophila* type IV secretion channel. *Nat. Microbiol.* 3 678–686. 10.1038/s41564-018-0165-z 29784975 PMC5970066

[B27] Christie P. J. (2016). The mosaic type IV secretion systems. *EcoSal Plus* 7:ecosalplus.ESP-0020-2015 10.1128/ecosalplus.ESP-0020-2015 PMC5119655 27735785

[B28] Christie P. J. Vogel J. P. (2000). Bacterial type IV secretion: conjugation systems adapted to deliver effector molecules to host cells. *Trends Microbiol.* 8 354–360. 10.1016/s0966-842x(00)01792-3 10920394 PMC4847720

[B29] Christie P. J. Whitaker N. González-Rivera C. (2014). Mechanism and structure of the bacterial type IV secretion systems. *Biochim. Biophys. Acta* 1843 1578–1591. 10.1016/j.bbamcr.2013.12.019 24389247 PMC4061277

[B30] Comas I. Moya A. Azad R. K. Lawrence J. G. Gonzalez-Candelas F. (2006). The evolutionary origin of Xanthomonadales genomes and the nature of the horizontal gene transfer process. *Mol. Biol. Evol.* 23 2049–2057. 10.1093/molbev/msl075 16882701

[B31] Costa T. R. D. Ilangovan A. Ukleja M. Redzej A. Santini J. M. Smith T. K. (2016). Structure of the bacterial sex F pilus reveals an assembly of a stoichiometric protein-phospholipid complex. *Cell* 166 1436–1444.e10. 10.1016/j.cell.2016.08.025 27610568 PMC5018250

[B32] Crossman L. C. Gould V. C. Dow J. M. Vernikos G. S. Okazaki A. Sebaihia M. (2008). The complete genome, comparative and functional analysis of *Stenotrophomonas maltophilia* reveals an organism heavily shielded by drug resistance determinants. *Genome Biol.* 9:R74. 10.1186/gb-2008-9-4-r74 18419807 PMC2643945

[B33] Da Silva A. C. R. Ferro J. A. Reinach F. C. Farah C. S. Furlan L. R. Quaggio R. B. (2002). Comparison of the genomes of two *Xanthomonas* pathogens with differing host specificities. *Nature* 417 459–463. 10.1038/417459a 12024217

[B34] de Bruijn I. Cheng X. de Jager V. Expósito R. G. Watrous J. Patel N. (2015). Comparative genomics and metabolic profiling of the genus *Lysobacter*. *BMC Genomics* 16:991. 10.1186/s12864-015-2191-z 26597042 PMC4657364

[B35] de la Cruz F. Davies J. (2000). Horizontal gene transfer and the origin of species: lessons from bacteria. *Trends Microbiol.* 8 128–133. 10.1016/s0966-842x(00)01703-0 10707066

[B36] Ding Z. Atmakuri K. Christie P. J. (2003). The outs and ins of bacterial type IV secretion substrates. *Trends Microbiol.* 11 527–535. 10.1016/j.tim.2003.09.004 14607070 PMC4844353

[B37] Durand E. Oomen C. Waksman G. (2010). Biochemical dissection of the ATPase TraB, the VirB4 homologue of the *Escherichia coli* pKM101 conjugation machinery. *J. Bacteriol.* 192 2315–2323. 10.1128/JB.01384-09 20172994 PMC2863485

[B38] Eisenbrandt R. Kalkum M. Lai E. M. Lurz R. Kado C. I. Lanka E. (1999). Conjugative pili of IncP plasmids, and the Ti plasmid T pilus are composed of cyclic subunits. *J. Biol. Chem.* 274 22548–22555. 10.1074/jbc.274.32.22548 10428832

[B39] El-Gebali S. Mistry J. Bateman A. Eddy S. R. Luciani A. Potter S. C. (2019). The Pfam protein families database in 2019. *Nucleic Acids Res.* 47 D427–D432. 10.1093/nar/gky995 30357350 PMC6324024

[B40] Ensminger A. W. Isberg R. R. (2009). *Legionella pneumophila* Dot/Icm translocated substrates: a sum of parts. *Curr. Opin. Microbiol.* 12 67–73. 10.1016/j.mib.2008.12.004 19157961 PMC2741304

[B41] Ferguson A. D. Amezcua C. A. Halabi N. M. Chelliah Y. Rosen M. K. Ranganathan R. (2007). Signal transduction pathway of TonB-dependent transporters. *Proc. Natl. Acad. Sci. U.S.A.* 104 513–518. 10.1073/pnas.0609887104 17197416 PMC1760641

[B42] Finsel I. Hilbi H. (2015). Formation of a pathogen vacuole according to *Legionella pneumophila*: how to kill one bird with many stones. *Cell. Microbiol.* 17 935–950. 10.1111/cmi.12450 25903720

[B43] Firczuk M. Bochtler M. (2007). Folds and activities of peptidoglycan amidases. *FEMS Microbiol. Rev.* 31 676–691. 10.1111/j.1574-6976.2007.00084.x 17888003

[B44] Frick-Cheng A. E. Pyburn T. M. Voss B. J. McDonald W. H. Ohi M. D. Cover T. L. (2016). Molecular and structural analysis of the *Helicobacter pylori* cag type IV secretion system core complex. *mBio* 7:e02001-15. 10.1128/mBio.02001-15 26758182 PMC4725015

[B45] Fronzes R. Schafer E. Wang L. Saibil H. R. Orlova E. V. Waksman G. (2009). Structure of a type IV secretion system core complex. *Science* 323 266–268. 10.1126/science.1166101 19131631 PMC6710095

[B46] Garcia-Herrero A. Vogel H. J. (2005). Nuclear magnetic resonance solution structure of the periplasmic signalling domain of the TonB-dependent outer membrane transporter FecA from *Escherichia coli*. *Mol. Microbiol.* 58 1226–1237. 10.1111/j.1365-2958.2005.04889.x 16313612

[B47] Garza I. Christie P. J. (2013). A putative transmembrane leucine zipper of *Agrobacterium* VirB10 is essential for T-Pilus biogenesis but not type IV secretion. *J. Bacteriol.* 195 3022–3034. 10.1128/JB.00287-13 23625845 PMC3697533

[B48] Ghosal D. Chang Y. W. Jeong K. C. Vogel J. P. Jensen G. J. (2017). *In situ* structure of the *Legionella* Dot/Icm type IV secretion system by electron cryotomography. *EMBO Rep.* 18 726–732. 10.15252/embr.201643598 28336774 PMC5412798

[B49] Gillespie J. J. Phan I. Q. H. Scheib H. Subramanian S. Edwards T. E. Lehman S. S. (2015). Structural insight into how bacteria prevent interference between multiple divergent type IV secretion systems. *mBio* 6:e01867-15. 10.1128/mBio.01867-15 26646013 PMC4676284

[B50] Gilmour M. W. Gunton J. E. Lawley T. D. Taylor D. E. (2003). Interaction between the IncHI1 plasmid R27 coupling protein and type IV secretion system: TraG associates with the coiled-coil mating pair formation protein TrhB. *Mol. Microbiol.* 49 105–116. 10.1046/j.1365-2958.2003.03551.x 12823814

[B51] Gomis-Ruth F. X. Moncalian G. de la Cruz F. Coll M. (2002). Conjugative plasmid protein TrwB, an integral membrane type IV secretion system coupling protein. Detailed structural features and mapping of the active site cleft. *J. Biol. Chem.* 277 7556–7566. 10.1074/jbc.m110462200 11748238

[B52] Gonzalez-Rivera C. Bhatty M. Christie P. J. (2016). Mechanism and function of type IV secretion during infection of the human host. *Microbiol. Spectr.* 4:VMBF-0024-2015. 10.1128/microbiolspec.VMBF-0024-2015 27337453 PMC4920089

[B53] Gordon J. E. Costa T. R. D. Patel R. S. Gonzalez-Rivera C. Sarkar M. K. Orlova E. V. (2017). Use of chimeric type IV secretion systems to define contributions of outer membrane subassemblies for contact-dependent translocation. *Mol. Microbiol.* 105 273–293. 10.1111/mmi.13700 28452085 PMC5518639

[B54] Grohmann E. Christie P. J. Waksman G. Backert S. (2017). Type IV secretion in Gram-negative and Gram-positive bacteria. *Mol. Microbiol.* 107 455–471. 10.1111/mmi.13896 29235173 PMC5796862

[B55] Guglielmini J. Neron B. Abby S. S. Garcillan-Barcia M. P. de la Cruz F. Rocha E. P. (2014). Key components of the eight classes of type IV secretion systems involved in bacterial conjugation or protein secretion. *Nucleic Acids Res.* 42 5715–5727. 10.1093/nar/gku194 24623814 PMC4027160

[B56] Hamilton H. L. Dominguez N. M. Schwartz K. J. Hackett K. T. Dillard J. P. (2005). *Neisseria gonorrhoeae* secretes chromosomal DNA via a novel type IV secretion system. *Mol. Microbiol.* 55 1704–1721. 10.1111/j.1365-2958.2005.04521.x 15752195

[B57] Hare S. Bayliss R. Baron C. Waksman G. (2006). A large domain swap in the VirB11 ATPase of *Brucella suis* leaves the hexameric assembly intact. *J. Mol. Biol.* 360 56–66. 10.1016/j.jmb.2006.04.060 16730027

[B58] Harms A. Liesch M. Körner J. Québatte M. Engel P. Dehio C. (2017). A bacterial toxin-antitoxin module is the origin of inter-bacterial and inter-kingdom effectors of *Bartonella*. *PLoS Genet.* 13:e1007077. 10.1371/journal.pgen.1007077 29073136 PMC5675462

[B59] Hayes C. S. Koskiniemi S. Ruhe Z. C. Poole S. J. Low D. A. (2014). Mechanisms and biological roles of contact-dependent growth inhibition systems. *Cold Spring Harb. Perspect. Med.* 4:a010025. 10.1101/cshperspect.a010025 24492845 PMC3904093

[B60] Hayward A. C. Fegan N. Fegan M. Stirling G. R. (2010). *Stenotrophomonas* and Lysobacter: ubiquitous plant-associated gamma-proteobacteria of developing significance in applied microbiology. *J. Appl. Microbiol.* 108 756–770. 10.1111/j.1365-2672.2009.04471.x 19702860

[B61] He Y. Q. Zhang L. Jiang B. L. Zhang Z. C. Xu R. Q. Tang D. J. (2007). Comparative and functional genomics reveals genetic diversity and determinants of host specificity among reference strains and a large collection of Chinese isolates of the phytopathogen *Xanthomonas campestris* pv. *campestris*. *Genome Biol.* 8:R218. 17927820 10.1186/gb-2007-8-10-r218 PMC2246292

[B62] Hilbi H. Nagai H. Kubori T. Roy C. R. (2017). Subversion of host membrane dynamics by the legionella Dot/Icm type IV secretion system. *Curr. Top. Microbiol. Immunol.* 413 221–242. 10.1007/978-3-319-75241-9_9 29536361

[B63] Hofreuter D. Odenbreit S. Haas R. (2001). Natural transformation competence in *Helicobacter pylori* is mediated by the basic components of a type IV secretion system. *Mol. Microbiol.* 41 379–391. 10.1046/j.1365-2958.2001.02502.x 11489125

[B64] Jakubowski S. J. Kerr J. E. Garza I. Krishnamoorthy V. Bayliss R. Waksman G. (2009). *Agrobacterium* VirB10 domain requirements for type IV secretion and T pilus biogenesis. *Mol. Microbiol.* 71 779–794. 10.1111/j.1365-2958.2008.06565.x 19054325 PMC3816096

[B65] Jakubowski S. J. Krishnamoorthy V. Cascales E. Christie P. J. (2004). *Agrobacterium tumefaciens* VirB6 domains direct the ordered export of a DNA substrate through a type IV secretion system. *J. Mol. Biol.* 341 961–977. 10.1016/j.jmb.2004.06.052 15328612 PMC3918220

[B66] Jensen R. B. Gerdes K. (1995). Programmed cell death in bacteria: proteic plasmid stabilization systems. *Mol. Microbiol.* 17 205–210. 10.1111/j.1365-2958.1995.mmi_17020205.x 7494469

[B67] Jones A. L. Shirasu K. Kado C. I. (1994). The product of the VirB4 gene of *Agrobacterium tumefaciens* promotes accumulation of VirB3 protein. *J. Bacteriol.* 176 5255–5261. 10.1128/jb.176.17.5255-5261.1994 8071199 PMC196708

[B68] Kanamaru S. Leiman P. G. Kostyuchenko V. A. Chipman P. R. Mesyanzhinov V. V. Arisaka F. (2002). Structure of the cell-puncturing device of bacteriophage T4. *Nature* 415 553–557. 10.1038/415553a 11823865

[B69] Kanehisa M. Furumichi M. Tanabe M. Sato Y. Morishima K. (2017). KEGG: new perspectives on genomes, pathways, diseases and drugs. *Nucleic Acids Res.* 45 D353–D361. 10.1093/nar/gkw1092 27899662 PMC5210567

[B70] Ke Y. Wang Y. Li W. Chen Z. (2015). Type IV secretion system of *Brucella* spp. and its effectors. *Front. Cell. Infect. Microbiol.* 5:72. 10.3389/fcimb.2015.00072 26528442 PMC4602199

[B71] Kondou Y. Kitazawa D. Takeda S. Tsuchiya Y. Yamashita E. Mizuguchi M. (2005). Structure of the central hub of bacteriophage Mu baseplate determined by X-ray crystallography of gp44. *J. Mol. Biol.* 352 976–985. 10.1016/j.jmb.2005.07.044 16125724

[B72] Korotkov K. V. Pardon E. Steyaert J. Hol W. G. (2009). Crystal structure of the N-terminal domain of the secretin GspD from ETEC determined with the assistance of a nanobody. *Structure* 17 255–265. 10.1016/j.str.2008.11.011 19217396 PMC2662362

[B73] Krogh A. Larsson B. von Heijne G. Sonnhammer E. L. (2001). Predicting transmembrane protein topology with a hidden Markov model: application to complete genomes. *J. Mol. Biol.* 305 567–580. 10.1006/jmbi.2000.4315 11152613

[B74] Kuroda T. Kubori T. Thanh Bui X. Hyakutake A. Uchida Y. Imada K. (2015). Molecular and structural analysis of *Legionella* DotI gives insights into an inner membrane complex essential for type IV secretion. *Sci. Rep.* 5:10912. 10.1038/srep10912 26039110 PMC4454188

[B75] Lederberg J. Tatum E. L. (1946). Gene recombination in *Escherichia coli*. *Nature* 158:558 10.1038/158558a0 21001945

[B76] Lee B. M. Park Y. J. Park D. S. Kang H. W. Kim J. G. Song E. S. (2005). The genome sequence of *Xanthomonas oryzae* pathovar oryzae KACC10331, the bacterial blight pathogen of rice. *Nucleic Acids Res.* 33 577–586. 10.1093/nar/gki206 15673718 PMC548351

[B77] Leiman P. G. Basler M. Ramagopal U. A. Bonanno J. B. Sauder J. M. Pukatzki S. (2009). Type VI secretion apparatus and phage tail-associated protein complexes share a common evolutionary origin. *Proc. Natl. Acad. Sci. U.S.A.* 106 4154–4159. 10.1073/pnas.0813360106 19251641 PMC2657435

[B78] Li Y. G. Christie P. J. (2018). The *Agrobacterium* VirB/VirD4 T4SS: mechanism and architecture defined through in vivo mutagenesis and chimeric systems. *Curr. Top. Microbiol. Immunol.* 418 233–260. 10.1007/82_2018_94 29808338 PMC7011205

[B79] Llosa M. Alkorta I. (2017). Coupling proteins in type IV secretion. *Curr. Top. Microbiol. Immunol.* 413 143–168. 10.1007/978-3-319-75241-9_6 29536358

[B80] Llosa M. Zunzunegui S. de la Cruz F. (2003). Conjugative coupling proteins interact with cognate and heterologous VirB10-like proteins while exhibiting specificity for cognate relaxosomes. *Proc. Natl. Acad. Sci. U.S.A.* 100 10465–10470. 10.1073/pnas.1830264100 12925737 PMC193584

[B81] Llosa M. Zupan J. Baron C. Zambryski P. (2000). The N- and C-terminal portions of the *Agrobacterium* VirB1 protein independently enhance tumorigenesis. *J. Bacteriol.* 182 3437–3445. 10.1128/jb.182.12.3437-3445.2000 10852875 PMC101919

[B82] Locht C. Coutte L. Mielcarek N. (2011). The ins and outs of pertussis toxin. *FEBS J.* 278 4668–4682. 10.1111/j.1742-4658.2011.08237.x 21740523

[B83] Looney W. J. Narita M. Mühlemann K. (2009). *Stenotrophomonas maltophilia*: an emerging opportunist human pathogen. *Lancet Infect. Dis.* 9 312–323. 10.1016/S1473-3099(09)70083-0 19393961

[B84] Low H. H. Gubellini F. Rivera-Calzada A. Braun N. Connery S. Dujeancourt A. (2014). Structure of a type IV secretion system. *Nature* 508 550–553. 10.1038/nature13081 24670658 PMC3998870

[B85] Mansfield J. Genin S. Magori S. Citovsky V. Sriariyanum M. Ronald P. (2012). Top 10 plant pathogenic bacteria in molecular plant pathology. *Mol. Plant Pathol.* 13 614–629. 10.1111/j.1364-3703.2012.00804.x 22672649 PMC6638704

[B86] Marchler-Bauer A. Derbyshire M. K. Gonzales N. R. Lu S. Chitsaz F. Geer L. Y. (2015). CDD: NCBI’s conserved domain database. *Nucleic Acids Res.* 43 D222–D226. 10.1093/nar/gku1221 25414356 PMC4383992

[B87] Martins-Pinheiro M. Galhardo R. S. Lage C. Lima-Bessa K. M. Aires K. A. Menck C. F. M. (2004). Different patterns of evolution for duplicated DNA repair genes in bacteria of the Xanthomonadales group. *BMC Evol. Biol.* 4:29. 10.1186/1471-2148-4-29 15333143 PMC518961

[B88] Moffatt J. H. Newton P. Newton H. J. (2015). *Coxiella burnetii*: turning hostility into a home. *Cell. Microbiol.* 17 621–631. 10.1111/cmi.12432 25728389

[B89] Moreira L. M. Almeida N. F. Jr. Potnis N. Digiampietri L. A. Adi S. S. Bortolossi J. C. (2010). Novel insights into the genomic basis of citrus canker based on the genome sequences of two strains of *Xanthomonas fuscans* subsp. *aurantifolii*. *BMC Genomics* 11:238. 10.1186/1471-2164-11-238 20388224 PMC2883993

[B90] Mukherjee P. Roy P. (2016). Genomic potential of *Stenotrophomonas maltophilia* in bioremediation with an assessment of its multifaceted role in our environment. *Front. Microbiol.* 7:967. 10.3389/fmicb.2016.00967 27446008 PMC4916776

[B91] Nakai K. Horton P. (1999). PSORT: a program for detecting sorting signals in proteins and predicting their subcellular localization. *Trends Biochem. Sci.* 24 34–35. 10.1016/S0968-0004(98)01336-X 10087920

[B92] Nakano N. Kubori T. Kinoshita M. Imada K. Nagai H. (2010). Crystal structure of *Legionella* DotD: insights into the relationship between type IVB and type II/III secretion systems. *PLoS Pathog.* 6:e1001129. 10.1371/journal.ppat.1001129 20949065 PMC2951367

[B93] Naumann M. Sokolova O. Tegtmeyer N. Backert S. (2017). *Helicobacter pylori*: a paradigm pathogen for subverting host cell signal transmission. *Trends Microbiol.* 25 316–328. 10.1016/j.tim.2016.12.004 28057411

[B94] Naushad H. S. Gupta R. S. (2013). Phylogenomics and molecular signatures for species from the plant pathogen-containing order Xanthomonadales. *PLoS One* 8:e55216. 10.1371/journal.pone.0055216 23408961 PMC3568101

[B95] Naushad S. Adeolu M. Wong S. Sohail M. Schellhorn H. E. Gupta R. S. (2015). A phylogenomic and molecular marker based taxonomic framework for the order *Xanthomonadales*: proposal to transfer the families *Algiphilaceae* and *Solimonadaceae* to the order *Nevskiales* ord. nov. and to create a new family within the order *Xanthomonadales*, the family *Rhodanobacteraceae* fam. nov., containing the genus *Rhodanobacter* and its closest relatives. *Antonie Van Leeuwenhoek* 107 467–485. 10.1007/s10482-014-0344-8 25481407

[B96] Ochiai H. Inoue V. Takeya M. Sasaki A. Kaku H. (2005). Genome sequence of *Xanthomonas oryzae* pv. *oryzae* suggests contribution of large numbers of effector genes and insertion sequences to its race diversity. *Jpn. Agric. Res. Q.* 39 275–287. 10.6090/jarq.39.275

[B97] Oliveira L. C. Souza D. P. Oka G. U. Lima F. da S. Oliveira R. J. (2016). VirB7 and VirB9 interactions are required for the assembly and antibacterial activity of a type IV secretion system. *Structure* 24 1707–1718. 10.1016/j.str.2016.07.015 27594685

[B98] Panthee S. Hamamoto H. Paudel A. Sekimizu K. (2016). *Lysobacter* species: a potential source of novel antibiotics. *Arch. Microbiol.* 198 839–845. 10.1007/s00203-016-1278-5 27541998

[B99] Paschos A. Patey G. Sivanesan D. Gao C. Bayliss R. Waksman G. (2006). Dimerization and interactions of *Brucella suis* VirB8 with VirB4 and VirB10 are required for its biological activity. *Proc. Natl. Acad. Sci. U.S.A.* 103 7252–7257. 10.1073/pnas.0600862103 16648257 PMC1464329

[B100] Peña A. Matilla I. Martín-Benito J. Valpuesta J. M. Carrascosa J. L. De La Cruz F. (2012). The hexameric structure of a conjugative VirB4 protein ATPase provides new insights for a functional and phylogenetic relationship with DNA translocases. *J. Biol. Chem.* 287 39925–39932. 10.1074/jbc.M112.413849 23035111 PMC3501061

[B101] Pieretti I. Royer M. Barbe V. Carrere S. Koebnik R. Cociancich S. (2009). The complete genome sequence of *Xanthomonas albilineans* provides new insights into the reductive genome evolution of the xylem-limited *Xanthomonadaceae*. *BMC Genomics* 10:616. 10.1186/1471-2164-10-616 20017926 PMC2810307

[B102] Qian W. Jia Y. Ren S. X. He Y. Q. Feng J. X. Lu L. F. (2005). Comparative and functional genomic analyses of the pathogenicity of phytopathogen *Xanthomonas campestris* pv. *campestris*. *Genome Res.* 15 757–767. 10.1101/gr.3378705 15899963 PMC1142466

[B103] Qiu J. Luo Z. Q. (2017). *Legionella* and *Coxiella* effectors: strength in diversity and activity. *Nat. Rev. Microbiol.* 15 591–605. 10.1038/nrmicro.2017.67 28713154

[B104] Rabel C. Grahn A. M. Lurz R. Lanka E. (2003). The VirB4 family of proposed traffic nucleoside triphosphatases: common motifs in plasmid RP4 TrbE are essential for conjugation and phage adsorption. *J. Bacteriol.* 185 1045–1058. 10.1128/JB.185.3.1045-1058.2003 12533481 PMC142825

[B105] Redzej A. Ukleja M. Connery S. Trokter M. Felisberto-Rodrigues C. Cryar A. (2017). Structure of a VirD4 coupling protein bound to a VirB type IV secretion machinery. *EMBO J.* 36 3080–3095. 10.15252/embj.201796629 28923826 PMC5916273

[B106] Ripoll-Rozada J. Zunzunegui S. de la Cruz F. Arechaga I. Cabezón E. (2013). Functional interactions of VirB11 traffic ATPases with VirB4 and VirD4 molecular motors in type IV secretion systems. *J. Bacteriol.* 195 4195–4201. 10.1128/JB.00437-13 23852869 PMC3754731

[B107] Rivera-Calzada A. Fronzes R. Savva C. G. Chandran V. Lian P. W. Laeremans T. (2013). Structure of a bacterial type IV secretion core complex at subnanometre resolution. *EMBO J.* 32 1195–1204. 10.1038/emboj.2013.58 23511972 PMC3630358

[B108] Russell A. B. Hood R. D. Bui N. K. LeRoux M. Vollmer W. Mougous J. D. (2011). Type VI secretion delivers bacteriolytic effectors to target cells. *Nature* 475 343–347. 10.1038/nature10244 21776080 PMC3146020

[B109] Russell A. B. LeRoux M. Hathazi K. Agnello D. M. Ishikawa T. Wiggins P. A. (2013). Diverse type VI secretion phospholipases are functionally plastic antibacterial effectors. *Nature* 496 508–512. 10.1038/nature12074 23552891 PMC3652678

[B110] Saddler G. S. Bradbury J. F. (2007). “Xanthomonadales ord. nov,” in *Bergey’s Manual of Systematic Bacteriology*, eds Brenner D. J. Krieg N. R. Staley J. T. Garrity G. M. Boone D. R. Vos P. (Boston, MA: Springer). 10.1007/0-387-28022-7_3

[B111] Salzberg S. Schatz M. Delcher A. Roberts M. Rabinowicz P. Inman J. (2007). *Xanthomonas oryzae* pv. oryzicola BLS256, whole genome shotgun sequencing project. Submitt. to GenBank database (AAQN00000000.1).

[B112] Salzberg S. L. Sommer D. D. Schatz M. C. Phillippy A. M. Rabinowicz P. D. Tsuge S. (2008). Genome sequence and rapid evolution of the rice pathogen *Xanthomonas oryzae* pv. *oryzae* PXO99A. *BMC Genomics* 9:204. 10.1186/1471-2164-9-204 18452608 PMC2432079

[B113] Schmidt-Eisenlohr H. Domke N. Angerer C. Wanner G. Zambryski P. C. Baron C. (1999). Vir proteins stabilize VirB5 and mediate its association with the T pilus of *Agrobacterium tumefaciens*. *J. Bacteriol.* 181 7485–7492. 10601205 10.1128/jb.181.24.7485-7492.1999 PMC94205

[B114] Segura R. L. Águila-Arcos S. Ugarte-Uribe B. Vecino A. J. De La Cruz F. Goñi F. M. (2013). The transmembrane domain of the T4SS coupling protein TrwB and its role in protein-protein interactions. *Biochim. Biophys. Acta* 1828 2015–2025. 10.1016/j.bbamem.2013.05.022 23735543

[B115] Sexton J. A. Vogel J. P. (2002). Type IVB secretion by intracellular pathogens. *Traffic* 3 178–185. 10.1034/j.1600-0854.2002.030303.x 11886588

[B116] Sgro G. G. Costa T. R. D. Cenens W. Souza D. P. Cassago A. Coutinho de Oliveira L. (2018). Cryo-EM structure of the bacteria-killing type IV secretion system core complex from *Xanthomonas citri*. *Nat. Microbiol.* 3 1429–1440. 10.1038/s41564-018-0262-z 30349081 PMC6264810

[B117] Siamer S. Dehio C. (2015). New insights into the role of *Bartonella* effector proteins in pathogenesis. *Curr. Opin. Microbiol.* 23 80–85. 10.1016/j.mib.2014.11.007 25461577

[B118] Sivanesan D. Hancock M. A. Villamil Giraldo A. M. Baron C. (2010). Quantitative analysis of VirB8-VirB9-VirB10 interactions provides a dynamic model of type IV secretion system core complex assembly. *Biochemistry* 49 4483–4493. 10.1021/bi902201y 20426418

[B119] Souza D. P. Andrade M. O. Alvarez-Martinez C. E. Arantes G. M. Farah C. S. Salinas R. K. (2011). A component of the Xanthomonadaceae type IV secretion system combines a VirB7 motif with a N0 domain found in outer membrane transport proteins. *PLoS Pathog.* 7:e1002031. 10.1371/journal.ppat.1002031 21589901 PMC3093366

[B120] Souza D. P. Oka G. U. Alvarez-Martinez C. E. Bisson-Filho A. W. Dunger G. Hobeika L. (2015). Bacterial killing via a type IV secretion system. *Nat. Commun.* 6:6453. 10.1038/ncomms7453 25743609

[B121] Spagnuolo J. Opalka N. Wen W. X. Gagic D. Chabaud E. Bellini P. (2010). Identification of the gate regions in the primary structure of the secretin pIV. *Mol. Microbiol.* 76 133–150. 10.1111/j.1365-2958.2010.07085.x 20149106

[B122] Spreter T. Yip C. K. Sanowar S. Andre I. Kimbrough T. G. Vuckovic M. (2009). A conserved structural motif mediates formation of the periplasmic rings in the type III secretion system. *Nat. Struct. Mol. Biol.* 16 468–476. 10.1038/nsmb.1603 19396170 PMC2860953

[B123] Studholme D. J. Kemen E. Maclean D. Schornack S. Aritua V. Thwaites R. (2010). Genome-wide sequencing data reveals virulence factors implicated in banana *Xanthomonas* wilt. *FEMS Microbiol. Lett.* 310 182–192. 10.1111/j.1574-6968.2010.02065.x 20695894

[B124] Terradot L. Bayliss R. Oomen C. Leonard G. A. Baron C. Waksman G. (2005). Structures of two core subunits of the bacterial type IV secretion system, VirB8 from *Brucella suis* and ComB10 from *Helicobacter pylori*. *Proc. Natl. Acad. Sci. U.S.A.* 102 4596–4601. 10.1073/pnas.0408927102 15764702 PMC555499

[B125] Thieme F. Koebnik R. Bekel T. Berger C. Boch J. Buttner D. (2005). Insights into genome plasticity and pathogenicity of the plant pathogenic bacterium *Xanthomonas campestris* pv. vesicatoria revealed by the complete genome sequence. *J. Bacteriol.* 187 7254–7266. 10.1128/JB.187.21.7254-7266.2005 16237009 PMC1272972

[B126] Tzfira T. Citovsky V. (2006). Agrobacterium-mediated genetic transformation of plants: biology and biotechnology. *Curr. Opin. Biotechnol.* 17 147–154. 10.1016/j.copbio.2006.01.009 16459071

[B127] Van Herreweghe J. M. Vanderkelen L. Callewaert L. Aertsen A. Compernolle G. Declerck P. J. (2010). Lysozyme inhibitor conferring bacterial tolerance to invertebrate type lysozyme. *Cell. Mol. Life Sci.* 67 1177–1188. 10.1007/s00018-009-0241-x 20049505 PMC11115509

[B128] Van Sluys M. A. Monteiro-Vitorello C. B. Camargo L. E. A. Menck C. F. M. da Silva A. C. Ferro J. A. (2002). Comparative genomic analysis of plant-associated bacteria. *Annu. Rev. Phytopathol.* 40 169–189. 10.1146/annurev.phyto.40.030402.090559 12147758

[B129] Villamil Giraldo A. M. Mary C. Sivanesan D. Baron C. (2015). VirB6 and VirB10 from the *Brucella* type IV secretion system interact via the N-terminal periplasmic domain of VirB6. *FEBS Lett.* 589 1883–1889. 10.1016/j.febslet.2015.05.051 26071378

[B130] Villamil Giraldo A. M. Sivanesan D. Carle A. Paschos A. Smith M. A. Plesa M. (2012). Type IV secretion system core component VirB8 from *Brucella* binds to the globular domain of VirB5 and to a periplasmic domain of VirB6. *Biochemistry* 51 3881–3890. 10.1021/bi300298v 22515661

[B131] Vorholter F. J. Schneiker S. Goesmann A. Krause L. Bekel T. Kaiser O. (2008). The genome of *Xanthomonas campestris* pv. campestris B100 and its use for the reconstruction of metabolic pathways involved in xanthan biosynthesis. *J. Biotechnol.* 134 33–45. 10.1016/j.jbiotec.2007.12.013 18304669

[B132] Waksman G. (2019). From conjugation to T4S systems in Gram-negative bacteria: a mechanistic biology perspective. *EMBO Rep.* 20:e47012. 10.15252/embr.201847012 30602585 PMC6362355

[B133] Wasukira A. Tayebwa J. Thwaites R. Paszkiewicz K. Aritua V. Kubiriba J. (2012). Genome-wide sequencing reveals two major sub-lineages in the genetically monomorphic pathogen *Xanthomonas campestris* pathovar musacearum. *Genes* 3 361–377. 10.3390/genes3030361 24704974 PMC3902798

[B134] Whitaker N. Chen Y. Jakubowski S. J. Sarkar M. K. Li F. Christie P. J. (2015). The all-alpha domains of coupling proteins from the *Agrobacterium tumefaciens* VirB/VirD4 and *Enterococcus faecalis* pCF10-encoded type IV secretion systems confer specificity to binding of cognate DNA substrates. *J. Bacteriol.* 197 2335–2349. 10.1128/JB.00189-15 25939830 PMC4524192

[B135] Yeo H. J. Savvides S. N. Herr A. B. Lanka E. Waksman G. (2000). Crystal structure of the hexameric traffic ATPase of the *Helicobacter pylori* type IV secretion system. *Mol. Cell* 6 1461–1472. 10.1016/s1097-2765(00)00142-8 11163218

[B136] Zhang L. Wang X. Yu M. Qiao Y. Zhang X. H. (2015). Genomic analysis of *Luteimonas abyssi* XH031(T): insights into its adaption to the subseafloor environment of South Pacific Gyre and ecological role in biogeochemical cycle. *BMC Genomics* 16:1092. 10.1186/s12864-015-2326-2 26690083 PMC4687298

