# Supplementary material for: Bacteria-Killing Type IV Secretion Systems
Source: Front Microbiol. 2019 May 21;10:1078. doi: 10.3389/fmicb.2019.01078 (PMC6536674; doi:10.3389/fmicb.2019.01078)
Supplement: Supplementary file 1 [file Data_Sheet_1.PDF]

Supplementary Information for

Bacteria-killing Type IV Secretion Systems

Germán G. Sgro[1a], Gabriel U. Oka[1a], Diorge P. Souza[1£], William Cenens[1], Ethel Bayer-Santos[1‡], Bruno Matsuyama[1], Natalia F. Bueno[1], Thiago Rodrigo dos Santos[1], Cristina E. Alvarez-Martinez[2], Roberto K. Salinas[1], Chuck S. Farah[1]*

[1]Departamento de Bioquímica, Instituto de Química, Universidade de São Paulo, Av. Prof. Lineu Prestes 748, São Paulo, SP, 05508-000, Brazil

[2]Departamento de Genética, Evolução, Microbiologia e Imunologia Instituto de Biologia, UNICAMP, Rua Monteiro Lobato 255, Campinas, SP, 13083-862, Brazil.

*Corresponding author: Dr. Chuck S. Farah, Departamento de Bioquímica, Instituto de Química, Universidade de São Paulo, Av. Prof. Lineu Prestes 748, São Paulo, SP, 05508-000, Brazil. E-mail: chsfarah@iq.usp.br, telephone: (+55-11) 3091-3326

[a]Contributed equally

[£]Current address: MRC Laboratory for Molecular Cell Biology, University College London, Gower Street, London WC1E 6BT, UK

[‡]Current address: Departamento de Microbiologia, Instituto de Ciências Biomédicas, Universidade de São Paulo, Av. Prof. Lineu Prestes 1374, São Paulo, SP 05508-900, Brazil

Supplementary Figures 1 to 12

Supp. Figure 1

Sgro et al. (2019)

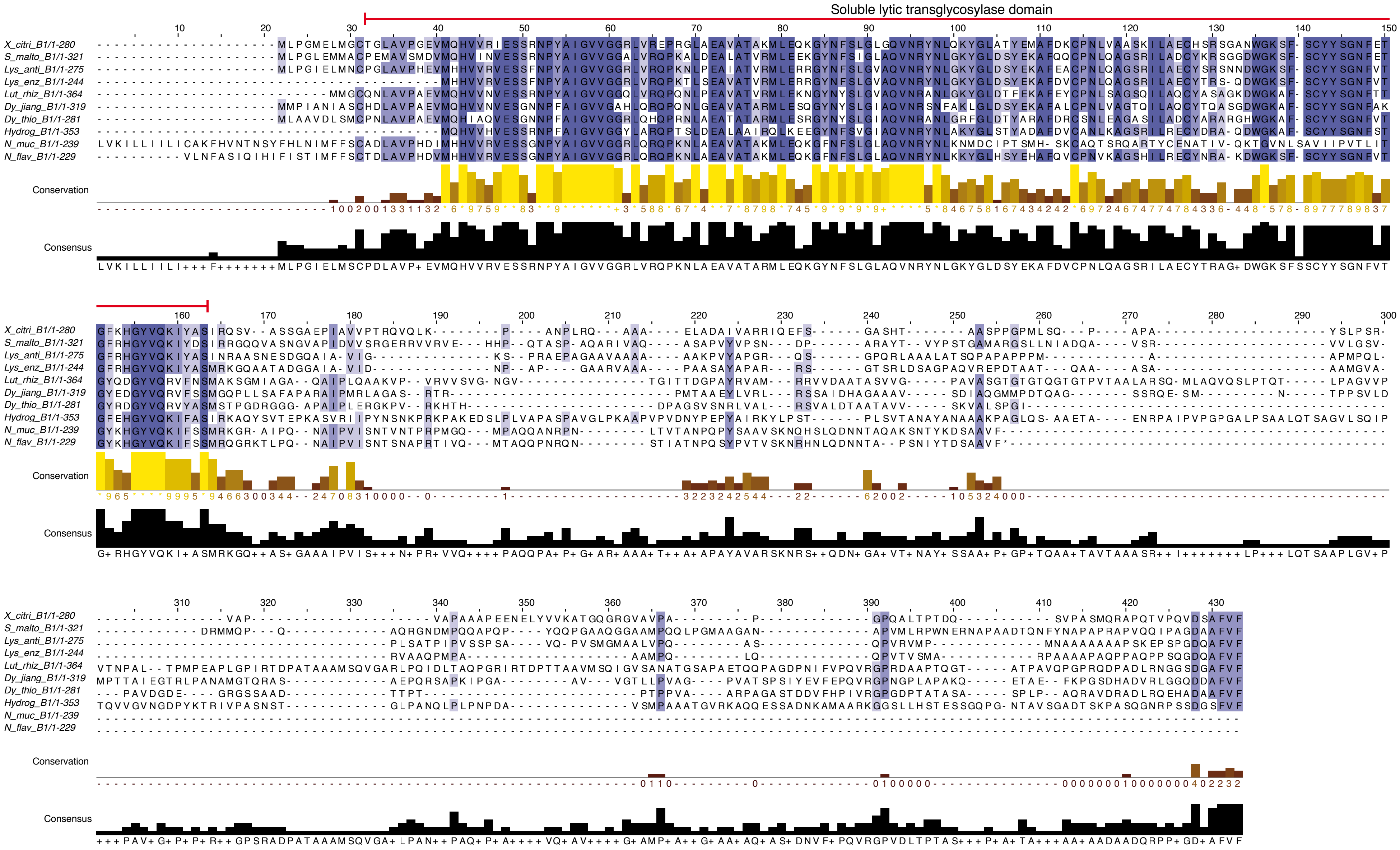

Supplementary Figure 1. X-T4SS VirB1 protein alignment. Multiple Sequence alignment of VirB1 proteins from the X-T4SSs of *Xanthomonas citri* 306 (X_citri; da Silva et al., 2002), *Stenotrophomonas maltophilia* K279a (S_malto; Crossman et al., 2008), *Lysobacter antibioticus* 76 (Lys_anti; de Bruijn et al., 2015), *Lysobacter enzymogenes* C3 (Lys_enz; unpublished; GenBank accession CP013140), *Luteibacter rhizovicinus* DSM16549 (Lut_rhiz; unpublished; GenBank accession CP017480), *Dyella jiangningensis* SBZ3-12 (Dy_jiang; Bao et al., 2014), *Dyella thiooxydans* ATSB10 (Dy_thio; unpublished; GenBank accession CP014841), *Hydrogenophaga crassostreae* LPB0072 (Hydrog; unpublished; GenBank accession LVWD01000013), *Neisseria mucosa* C102 (N_muc; unpublished, GenBank accession GCA_000186165) and *Neisseria flavescens* SK114 (N_flav; unpublished; GenBank accession ACQV01000009). The soluble lytic transglycosylase domain is indicated and terminates at a highly conserved Ser residue. The position of this Ser residue corresponds precisely with the cleavage site of VirB1 proteins from *A. tumefaciens* strains that result in the production of the VirB1* fragment that is subsequently secreted in that species (Zupan et al., 2007). Alignments were performed using Clustal Omega (McWilliam et al., 2013) and Figures were produced with JalView (Waterhouse et al., 2009).

Supp. Figure 2 Sgro et al. (2019)

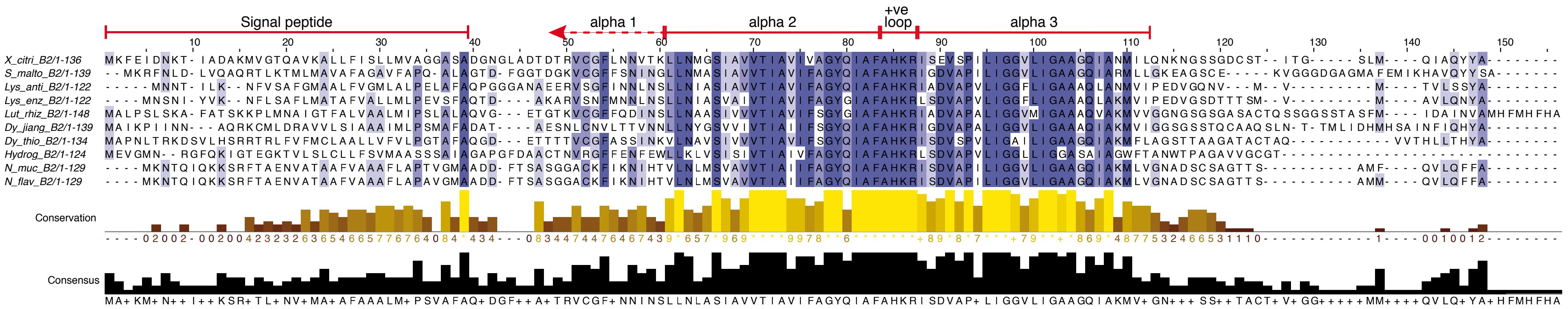

Supplementary Figure 2. X-T4SS VirB2 protein alignment. Multiple Sequence alignment of VirB2 proteins from the X-T4SSs of *Xanthomonas citri* 306 (X_citri; da Silva et al., 2002), *Stenotrophomonas maltophilia* K279a (S_malto; Crossman et al., 2008), *Lysobacter antibioticus* 76 (Lys_anti; de Bruijn et al., 2015), *Lysobacter enzymogenes* C3 (Lys_enz; unpublished; GenBank accession CP013140), *Luteibacter rhizovicinus* DSM16549 (Lut_rhiz; unpublished; GenBank accession CP017480), *Dyella jiangningensis* SBZ3-12 (Dy_jiang; Bao et al., 2014), *Dyella thiooxydans* ATSB10 (Dy_thio; unpublished; GenBank accession CP014841), *Hydrogenophaga crassostreae* LPB0072 (Hydrog; unpublished; GenBank accession LVWD01000013), *Neisseria mucosa* C102 (N_muc; unpublished, GenBank accession GCA_000186165) and *Neisseria flavescens* SK114 (N_flav; unpublished; GenBank accession ACQV01000009). Alignments were performed using Clustal Omega (McWilliam et al., 2013) and Figures were produced with JalView (Waterhouse et al., 2009). Analysis of these X-T4SS VirB2 protein sequences using the NetSurfP-2.0 algorithm (Klausen et al., 2019) predicts the presence of two central helices separated by a positively charged Ala-His-Lys-Arg (AHKR) loop. The first predicted helix corresponds to helices alpha 1 and 2 in the F and pED208 sex pili structures and the second helix likewise corresponds to alpha 3 in those structures (Costa et al., 2016). The secondary structure predictions also suggest the presence of a helical segment in the second half of the C-terminal extensions of all of the X-T4SS VirB2 proteins except for that from *H. crassostreae* whose C-terminal extension is significantly shorter (not shown due to the dispersion in Clustal Omega sequence alignments in this region). The conserved AHKR motif in X-T4SS VirB2 sequence (+ve loop) maps onto the positively charged loop between α helices 2 and 3 in the structures of the F and pED208 sex pili (Costa et al., 2016). In the F and pED208 sex pili structures, the loop residues interact with the head groups of bound phospholipids in the pilus lumen (Costa et al., 2016). Signal peptide prediction was performed using SignalP software (Petersen et al., 2011). Signal peptide cleavage is predicted to occur at the conserved Ala residue.

Supp. Figure 3

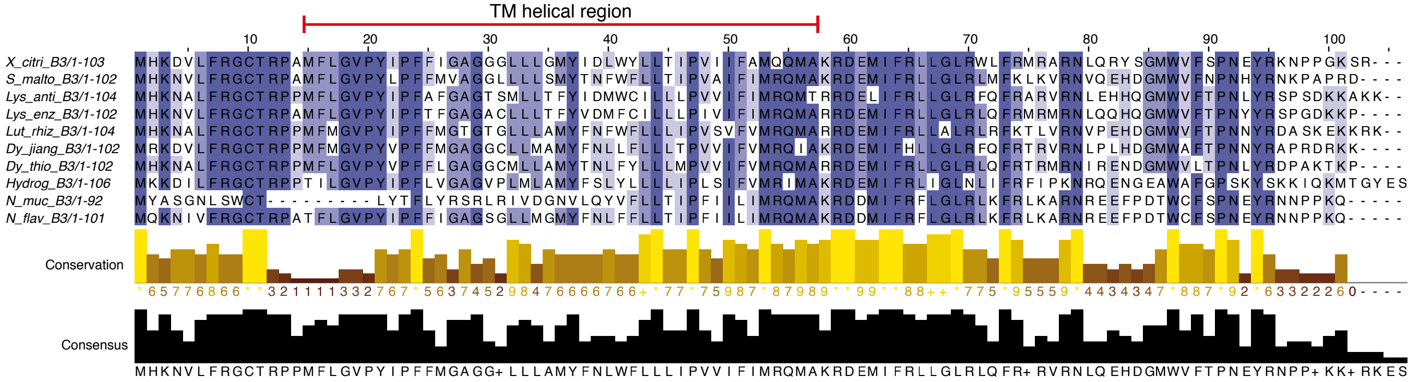

Supplementary Figure 3. X-T4SS VirB3 protein alignment. Multiple Sequence alignment of VirB3 proteins from the X-T4SSs of *Xanthomonas citri* 306 (X_citri; da Silva et al., 2002), *Stenotrophomonas maltophilia* K279a (S_malto; Crossman et al., 2008), *Lysobacter antibioticus* 76 (Lys_anti; de Bruijn et al., 2015), *Lysobacter enzymogenes* C3 (Lys_enz; unpublished; GenBank accession CP013140), *Luteibacter rhizovicinus* DSM16549 (Lut_rhiz; unpublished; GenBank accession CP017480), *Dyella jiangningensis* SBZ3-12 (Dy_jiang; Bao et al., 2014), *Dyella thiooxydans* ATSB10 (Dy_thio; unpublished; GenBank accession CP014841), *Hydrogenophaga crassostreae* LPB0072 (Hydrog; unpublished; GenBank accession LVWD01000013), *Neisseria mucosa* C102 (N_muc; unpublished, GenBank accession GCA_000186165) and *Neisseria flavescens* SK114 (N_flav; unpublished; GenBank accession ACQV01000009). Prediction of transmembrane helices using a variety of algorithms points to either one (HMMTop, TMpred, TMHMM, PSORT) or two (TMHMM, TOPCONS, PredictProtein, PSORT) TM helices for these proteins, all found in the region between residues 15 and 57. Therefore, the precise topology of the X-T4SS VirB3 proteins within the inner membrane is not at the moment clear. Transmembrane helix predictions were performed using the following algorithms: PredictProtein (Rost et al., 2007), HMMTop (Tusnády and Simon, 2001), TMpred (Hofmann and Stoffel, 1993), TMHMM (Krogh et al., 2001), TOPCONS (Tsirigos et al., 2015) and PSORT (Nakai and Horton, 1999). Sequence alignments were performed using Clustal Omega (McWilliam et al., 2013) and Figures were produced with JalView (Waterhouse et al., 2009).

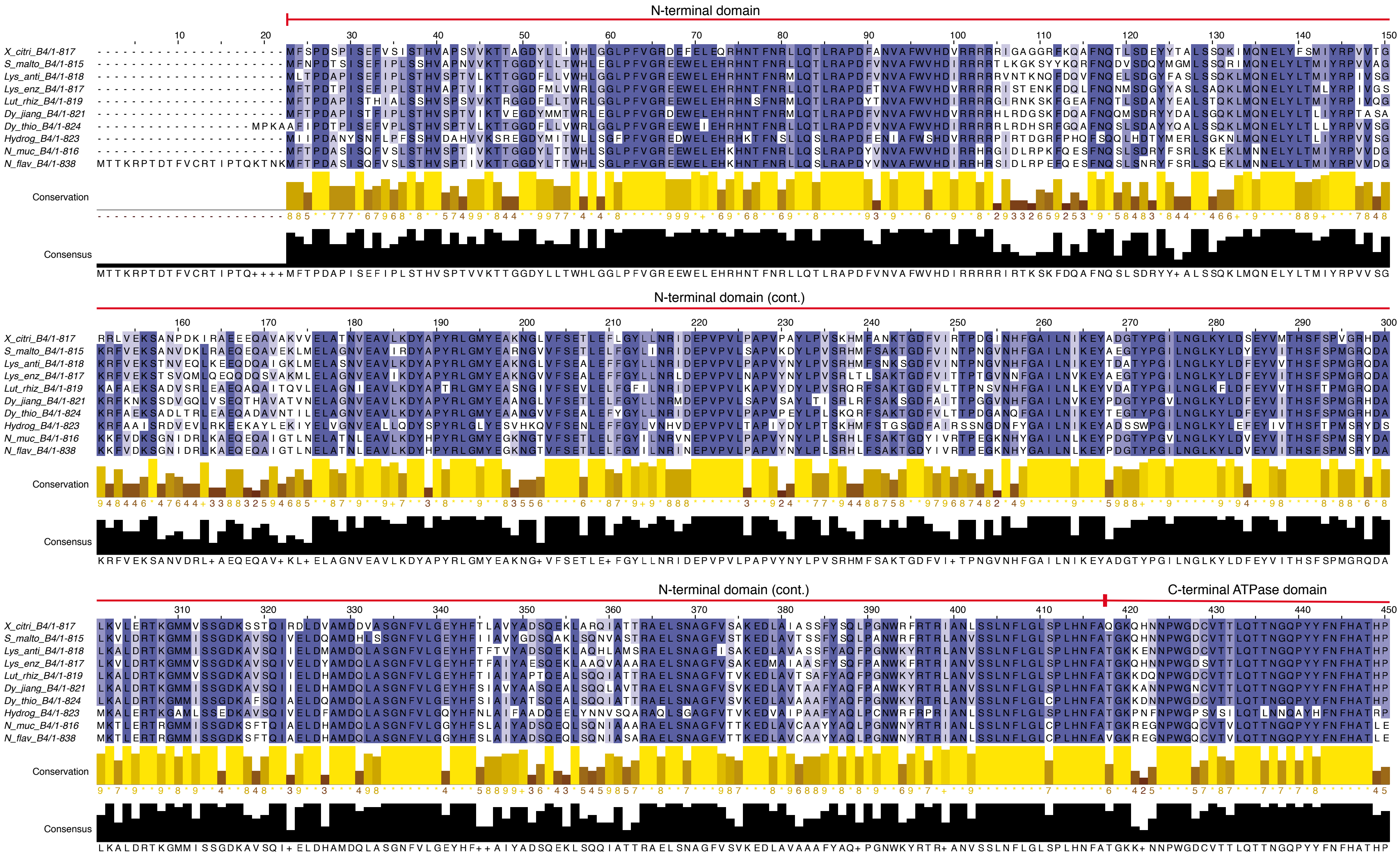
N-terminal domain
X_citri_B4/1-817
S_malto_B4/1-815
Lys_anti_B4/1-818
Lys_enz_B4/1-817
Lut_rhiz_B4/1-819
Dy_jiang_B4/1-821
Dy_thio_B4/1-824
Hydrog_B4/1-823
N_muc_B4/1-816
N_flav_B4/1-838
Conservation
Consensus
N-terminal domain (cont.)
N-terminal domain (cont.)
C-terminal ATPase domain

Supp. Figure 4 (cont.)

Sgro et al. (2019)

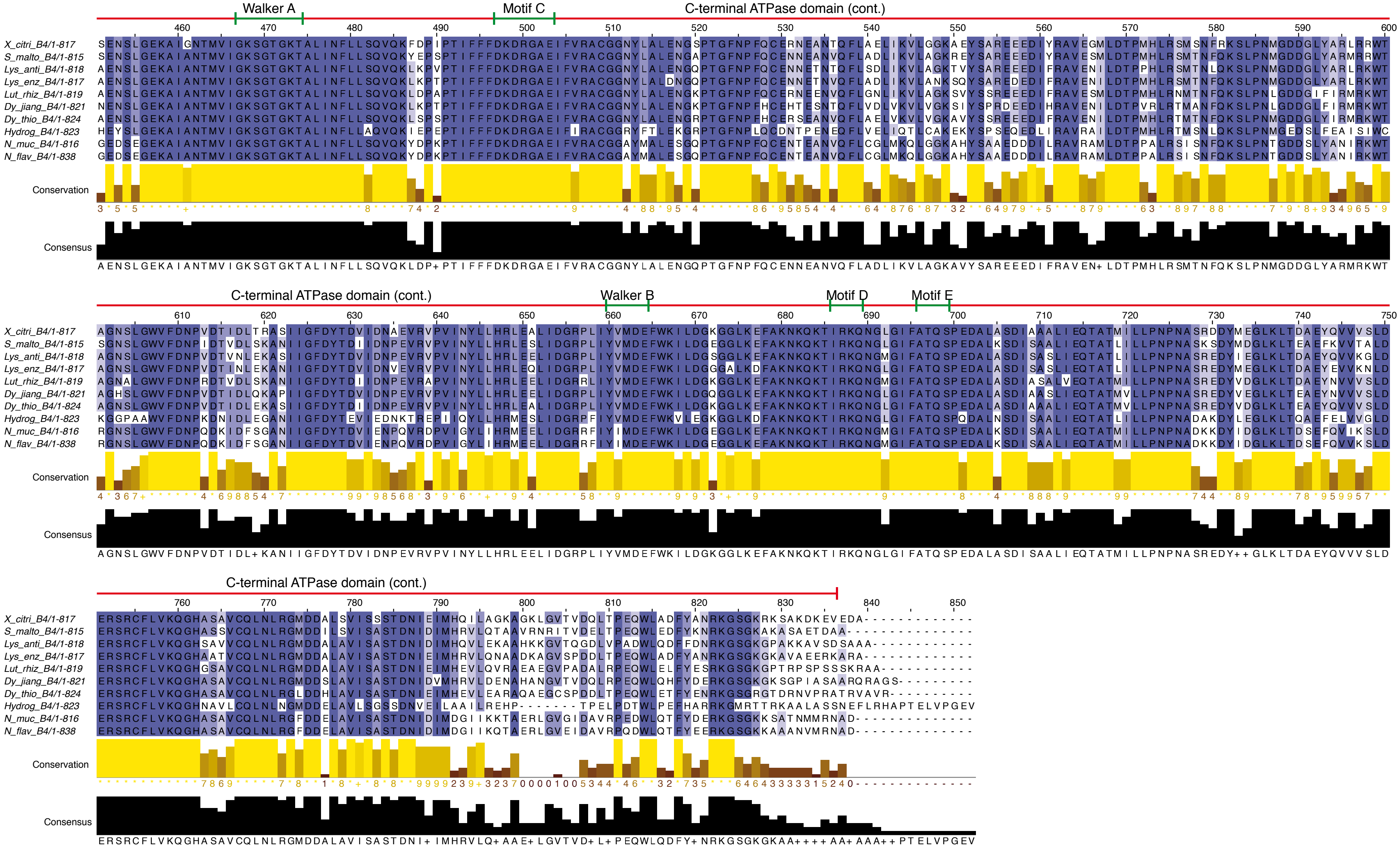

Supplementary Figure 4. X-T4SS VirB4 protein alignment. Multiple Sequence alignment of VirB4 proteins from the X-T4SSs of *Xanthomonas citri* 306 (X_citri; da Silva et al., 2002), *Stenotrophomonas maltophilia* K279a (S_malto; Crossman et al., 2008), *Lysobacter antibioticus* 76 (Lys_anti; de Bruijn et al., 2015), *Lysobacter enzymogenes* C3 (Lys_enz; unpublished; GenBank accession CP013140), *Luteibacter rhizovicinus* DSM16549 (Lut_rhiz; unpublished; GenBank accession CP017480), *Dyella jiangningensis* SBZ3-12 (Dy_jiang; Bao et al., 2014), *Dyella thiooxydans* ATSB10 (Dy_thio; unpublished; GenBank accession CP014841), *Hydrogenophaga crassostreae* LPB0072 (Hydrog; unpublished; GenBank accession LVWD01000013), *Neisseria mucosa* C102 (N_muc; unpublished, GenBank accession GCA_000186165) and *Neisseria flavescens* SK114 (N_flav; unpublished; GenBank accession ACQV01000009). Walker A and Walker B boxes and motifs C, D, and E were identified with reference to the *Thermoanaerobacter pseudethanolicus* VirD4 structure described in Wallden et al. (2012). Alignments were performed using Clustal Omega (McWilliam et al., 2013) and Figures were produced with JalView (Waterhouse et al., 2009).

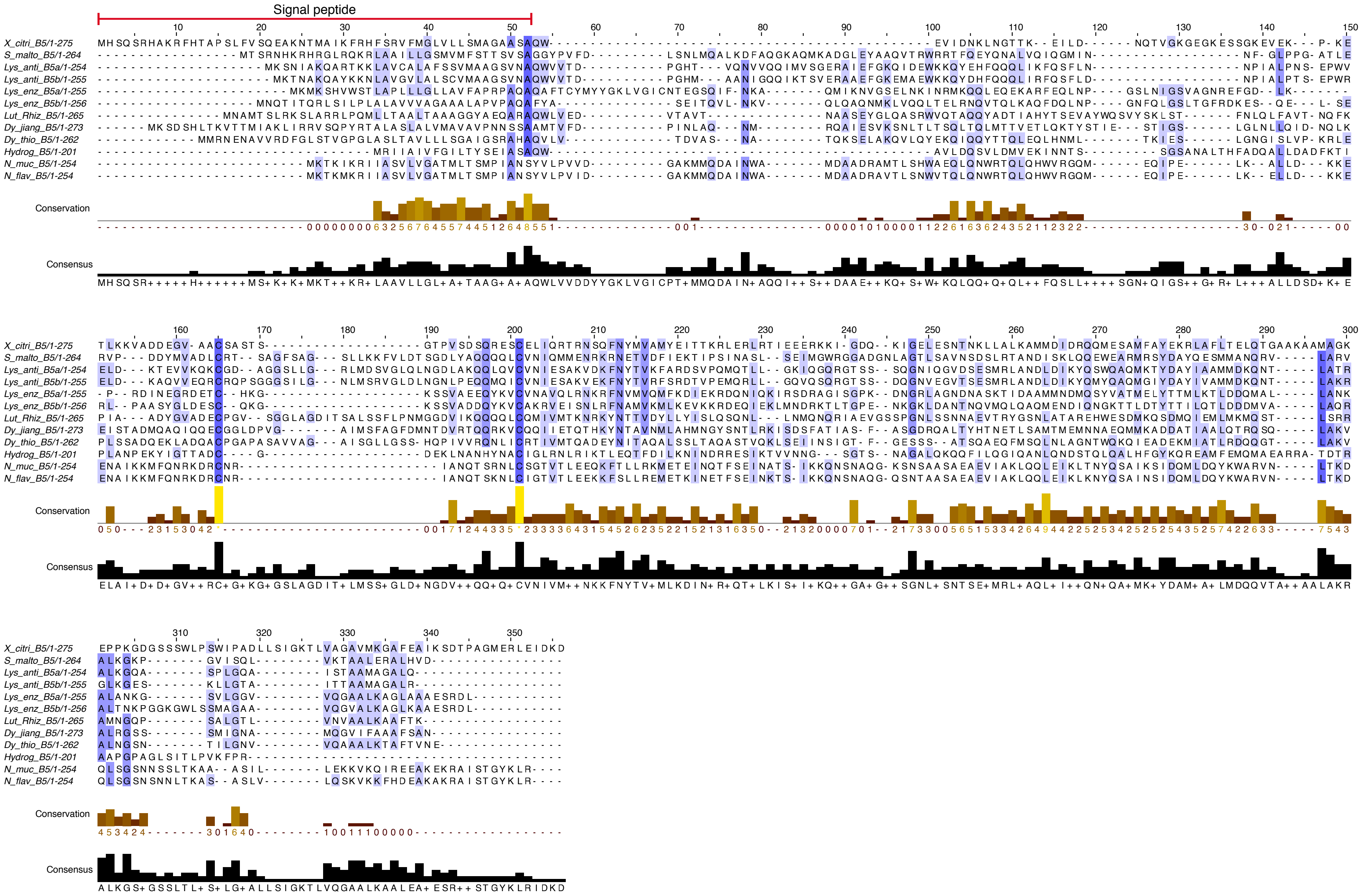
Signal peptide
X_citri_B5/1-275
S_malto_B5/1-264
Lys_anti_B5a/1-254
Lys_anti_B5b/1-255
Lys_enz_B5a/1-255
Lys_enz_B5b/1-256
Lut_Rhiz_B5/1-265
Dy_jiang_B5/1-273
Dy_thio_B5/1-262
Hydrog_B5/1-201
N_muc_B5/1-254
N_flav_B5/1-254
Conservation
Consensus

Supplementary Figure 5. X-T4SS VirB5 protein alignment. Multiple Sequence alignment of VirB5 proteins from the X-T4SSs of *Xanthomonas citri* 306 (X_citri; da Silva et al., 2002), *Stenotrophomonas maltophilia* K279a (S_malto; Crossman et al., 2008), *Lysobacter antibioticus* 76 (Lys_anti; de Bruijn et al., 2015), *Lysobacter enzymogenes* C3 (Lys_enz; unpublished; GenBank accession CP013140), *Luteibacter rhizovicinus* DSM16549 (Lut_rhiz; unpublished; GenBank accession CP017480), *Dyella jiangningensis* SBZ3-12 (Dy_jiang; Bao et al., 2014), *Dyella thiooxydans* ATSB10 (Dy_thio; unpublished; GenBank accession CP014841), *Hydrogenophaga crassostreae* LPB0072 (Hydrog; unpublished; GenBank accession LVWD01000013), *Neisseria mucosa* C102 (N_muc; unpublished, GenBank accession GCA_000186165) and *Neisseria flavescens* SK114 (N_flav; unpublished; GenBank accession ACQV01000009). Note the two absolutely conserved cysteine residues separated by a loop of variable length. Alignments were performed using Clustal Omega (McWilliam et al., 2013) and then manually adjusted to align the putative signal peptide cleavage sites as predicted by the SignalP algorithm (Petersen et al., 2011). Figures were produced with JalView (Waterhouse et al., 2009).

Supp. Figure 6

Sgro et al. (2019)

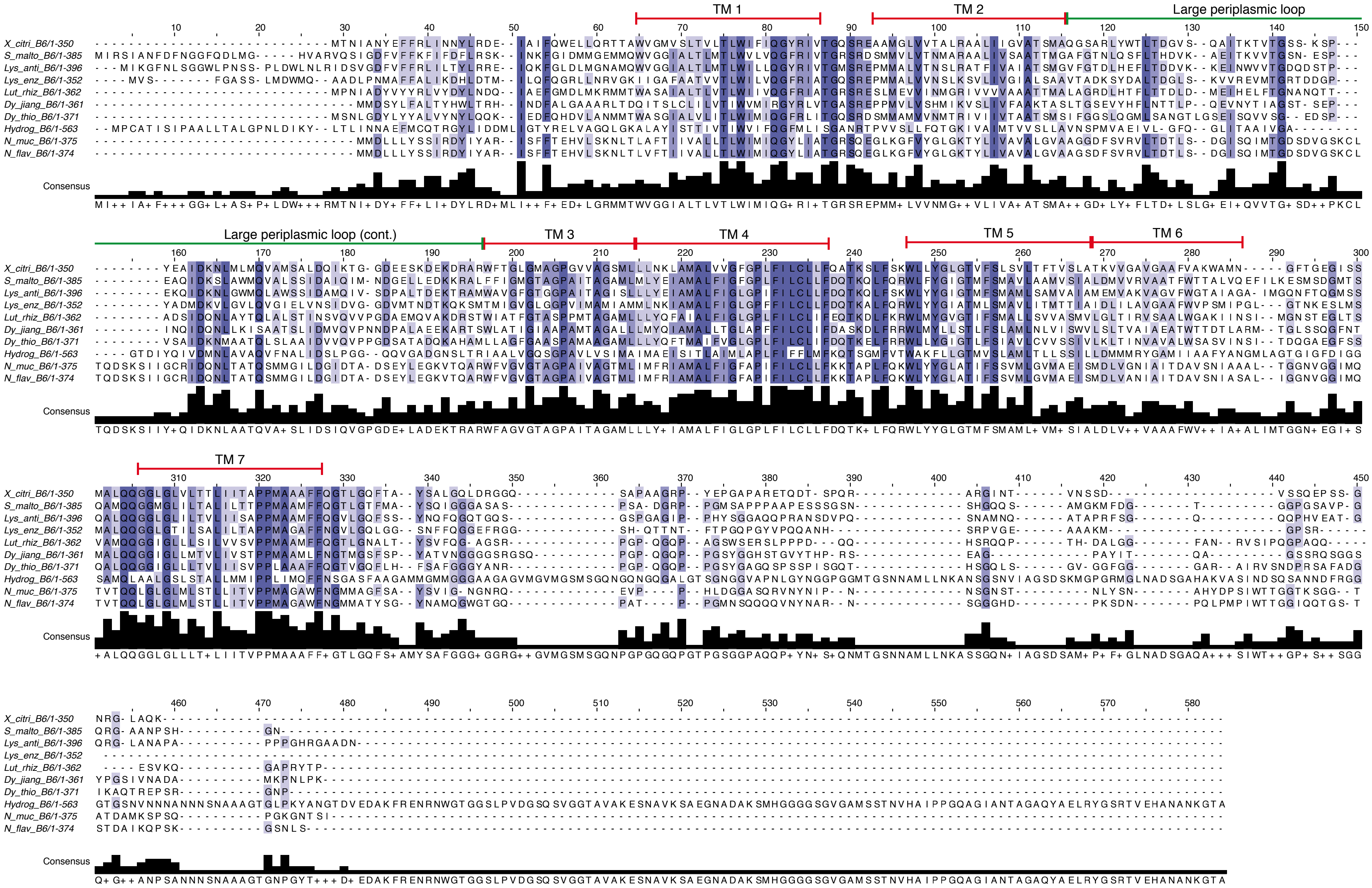

Supplementary Figure 6. X-T4SS VirB6 protein alignment. Multiple Sequence alignment of VirB6 proteins from the X-T4SSs of *Xanthomonas citri* 306 (X_citri; da Silva et al., 2002), *Stenotrophomonas maltophilia* K279a (S_malto; Crossman et al., 2008), *Lysobacter antibioticus* 76 (Lys_anti; de Bruijn et al., 2015), *Lysobacter enzymogenes* C3 (Lys_enz; unpublished; GenBank accession CP013140), *Luteibacter rhizovicinus* DSM16549 (Lut_rhiz; unpublished; GenBank accession CP017480), *Dyella jiangningensis* SBZ3-12 (Dy_jiang; Bao et al., 2014), *Dyella thiooxydans* ATSB10 (Dy_thio; unpublished; GenBank accession CP014841), *Hydrogenophaga crassostreae* LPB0072 (Hydrog; unpublished; GenBank accession LVWD01000013), *Neisseria mucosa* C102 (N_muc; unpublished, GenBank accession GCA_000186165) and *Neisseria flavescens* SK114 (N_flav; unpublished; GenBank accession ACQV01000009). Analysis of the VirB6 sequences using a several different algorithms (HMMTop, TMpred, TMHMM, PSORT, TOPCONS, PredictProtein) predicted 5, 6 or 7 transmembrane (TM) helices for these proteins. For convenience, seven putative transmembrane helices are indicated for the sequences most often identified by these algorithms. We note that the juxtaposition of transmembrane helix pairs 3/4 and 5/6 could be indicative of a single transmembrane helix in these regions. Therefore, the precise topology of the X-T4SS VirB6 proteins within the inner membrane is not at the moment clear. Transmembrane helix predictions were performed using the following algorithms: PredictProtein (Rost et al., 2007), HMMTop (Tusnády and Simon, 2001), TMpred (Hofmann and Stoffel, 1993), TMHMM (Krogh et al., 2001), TOPCONS (Tsirigos et al., 2015) and PSORT (Nakai and Horton, 1999). Alignments were performed using Clustal Omega (McWilliam et al., 2013) and Figures were produced with JalView (Waterhouse et al., 2009).

Supp. Figure 7

Sgro et al. (2019)

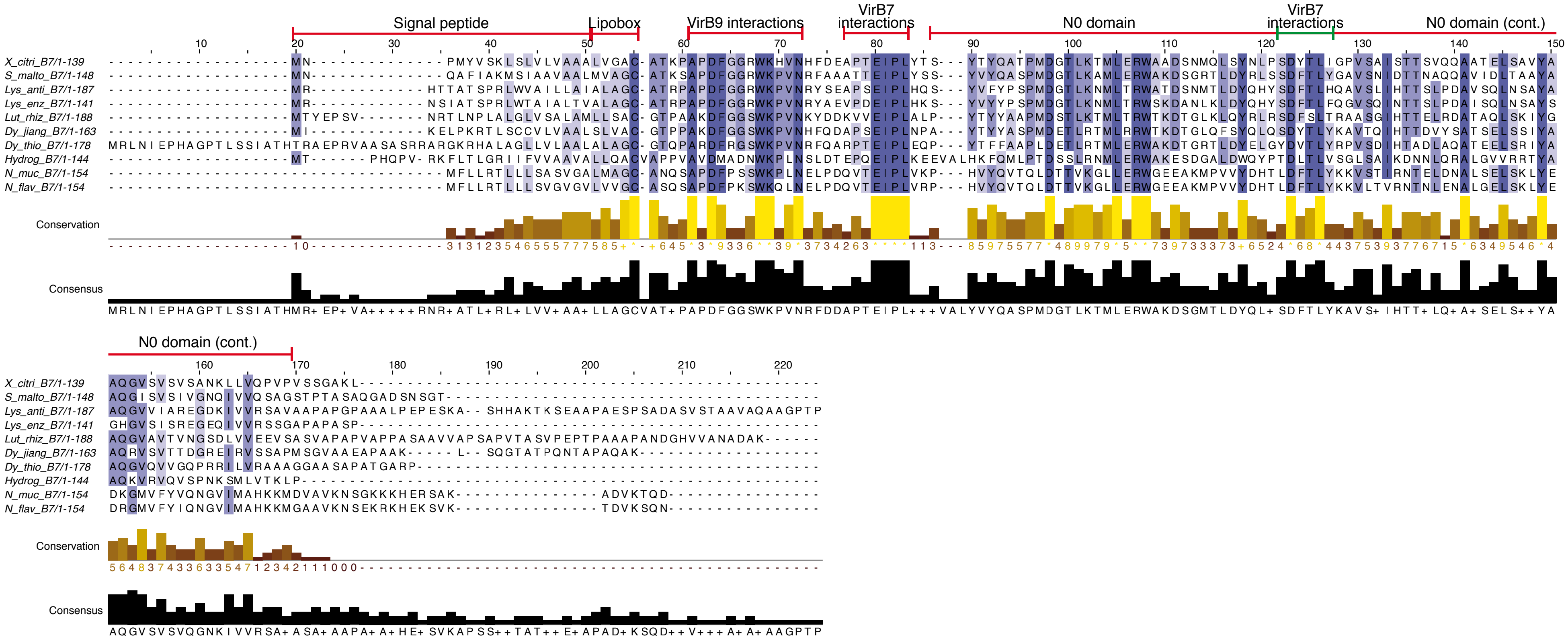

Supplementary Figure 7. X-T4SS VirB7 protein alignment. Multiple Sequence alignment of VirB7 proteins from the X-T4SSs of *Xanthomonas citri* 306 (X_citri; da Silva et al., 2002), *Stenotrophomonas maltophilia* K279a (S_malto; Crossman et al., 2008), *Lysobacter antibioticus* 76 (Lys_anti; de Bruijn et al., 2015), *Lysobacter enzymogenes* C3 (Lys_enz; unpublished; GenBank accession CP013140), *Luteibacter rhizovicinus* DSM16549 (Lut_rhiz; unpublished; GenBank accession CP017480), *Dyella jiangningensis* SBZ3-12 (Dy_jiang; Bao et al., 2014), *Dyella thiooxydans* ATSB10 (Dy_thio; unpublished; GenBank accession CP014841), *Hydrogenophaga crassostreae* LPB0072 (Hydrog; unpublished; GenBank accession LVWD01000013), *Neisseria mucosa* C102 (N_muc; unpublished, GenBank accession GCA_000186165) and *Neisseria flavescens* SK114 (N_flav; unpublished; GenBank accession ACQV01000009). The globular N0 domain and regions involved in VirB7-VirB9 and VirB7-VirB7 interactions are indicated, based on studies on the VirB7 protein of *X. citri* (Oliveira et al., 2016; Sgro et al., 2018; Souza et al., 2011). The N-terminal signal peptide for export into the periplasm and Lipobox are also indicated. Note that the *D. thiooxydans* VirB7 protein is annotated with an N-terminal extension and that a possible alternative start codon (Val26) would produce a polypeptide with a lipoprotein secretion signal similar to the other VirB7 proteins. Alignments were performed using Clustal Omega (McWilliam et al., 2013) and Figures were produced with JalView (Waterhouse et al., 2009).

Supp. Figure 8

Sgro et al. (2019)

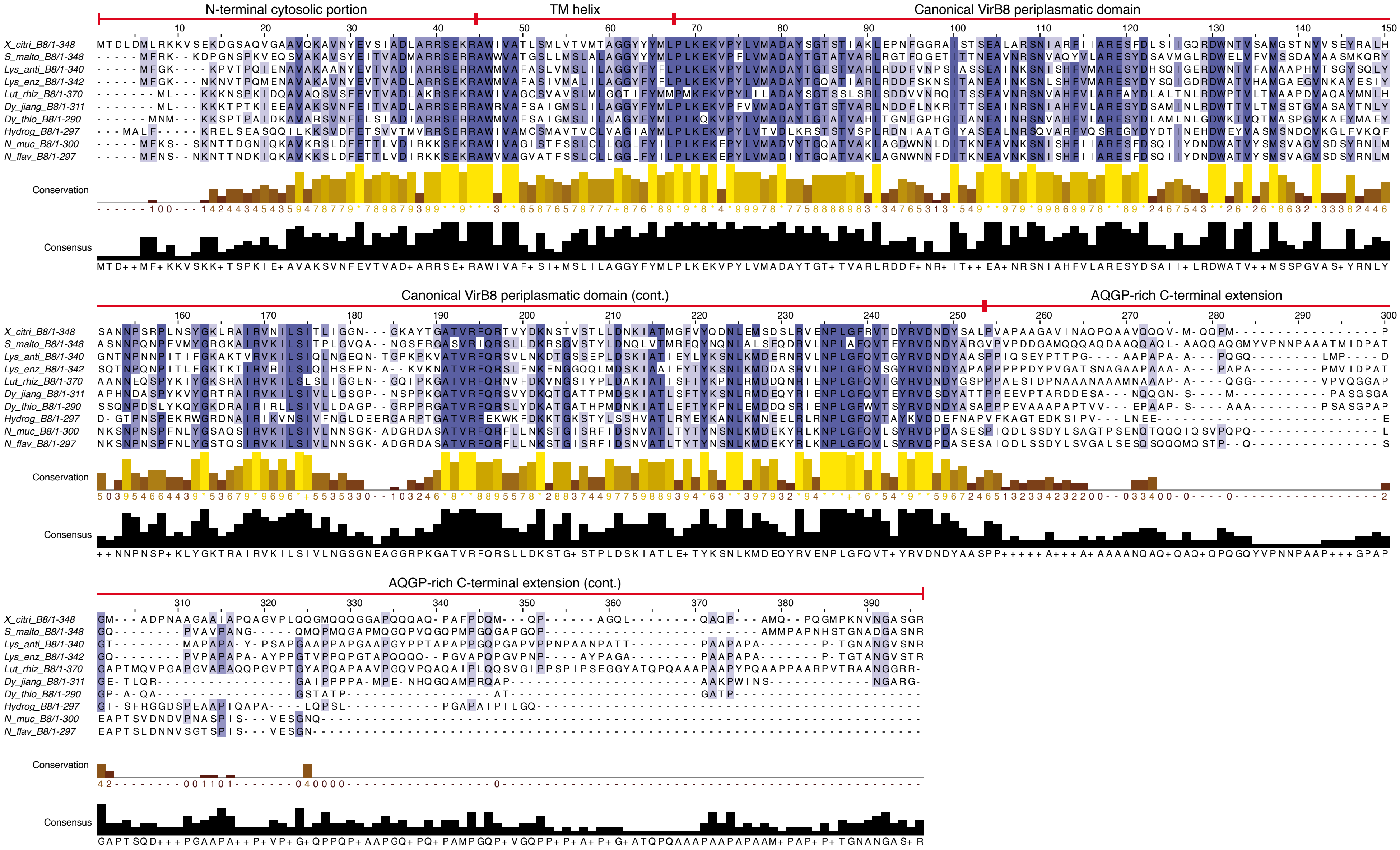

Supplementary Figure 8. X-T4SS VirB8 protein alignment. Multiple Sequence alignment of VirB8 proteins from the X-T4SSs of *Xanthomonas citri* 306 (X_citri; da Silva et al., 2002), *Stenotrophomonas maltophilia* K279a (S_malto; Crossman et al., 2008), *Lysobacter antibioticus* 76 (Lys_anti; de Bruijn et al., 2015), *Lysobacter enzymogenes* C3 (Lys_enz; unpublished; GenBank accession CP013140), *Luteibacter rhizovicinus* DSM16549 (Lut_rhiz; unpublished; GenBank accession CP017480), *Dyella jiangningensis* SBZ3-12 (Dy_jiang; Bao et al., 2014), *Dyella thiooxydans* ATSB10 (Dy_thio; unpublished; GenBank accession CP014841), *Hydrogenophaga crassostreae* LPB0072 (Hydrog; unpublished; GenBank accession LVWD01000013), *Neisseria mucosa* C102 (N_muc; unpublished, GenBank accession GCA_000186165) and *Neisseria flavescens* SK114 (N_flav; unpublished; GenBank accession ACQV01000009). VirB8 proteins from X-T4SSs have a sequence that aligns well with the VirB8 periplasmic domains from many other sources (see main text). X-T4SS VirB8 proteins all have C-terminal extensions that are enriched in Ala, Glu, Gly and Pro residues (AQGP-rich C-terminal extension; see Table 2). Analysis of the VirB8 sequences using several different algorithms all predicted a single transmembrane (TM) helix for these proteins. Transmembrane helix predictions were performed using the following algorithms: PredictProtein (Rost et al., 2007), HMMTop (Tusnády and Simon, 2001), TMpred (Hofmann and Stoffel, 1993), TMHMM (Krogh et al., 2001), TOPCONS (Tsirigos et al., 2015) and PSORT (Nakai and Horton, 1999). Alignments were performed using Clustal Omega (McWilliam et al., 2013) and Figures were produced with JalView (Waterhouse et al., 2009).

Supp. Figure 9

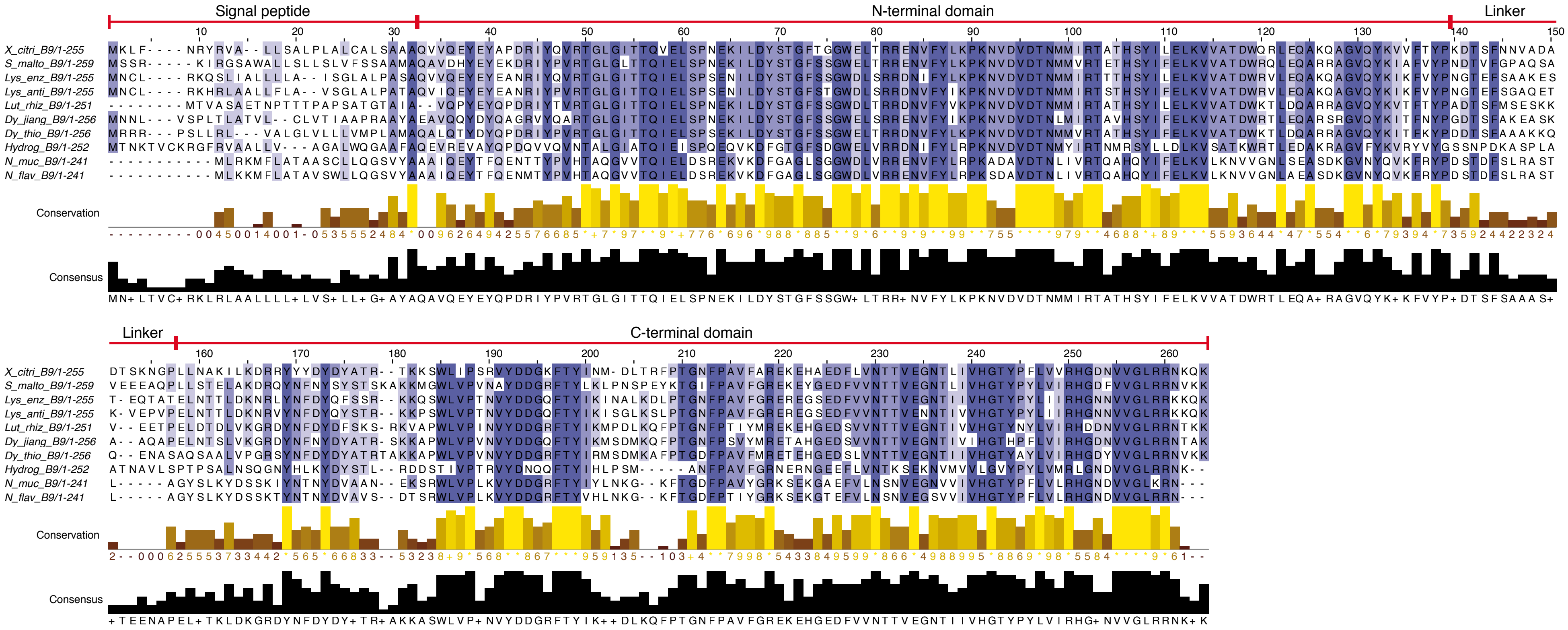

Supplementary Figure 9. X-T4SS VirB9 protein alignment. Multiple Sequence alignment of VirB9 proteins from the X-T4SSs of *Xanthomonas citri* 306 (X_citri; da Silva et al., 2002), *Stenotrophomonas maltophilia* K279a (S_malto; Crossman et al., 2008), *Lysobacter antibioticus* 76 (Lys_anti; de Bruijn et al., 2015), *Lysobacter enzymogenes* C3 (Lys_enz; unpublished; GenBank accession CP013140), *Luteibacter rhizovicinus* DSM16549 (Lut_rhiz; unpublished; GenBank accession CP017480), *Dyella jiangningensis* SBZ3-12 (Dy_jiang; Bao et al., 2014), *Dyella thiooxydans* ATSB10 (Dy_thio; unpublished; GenBank accession CP014841), *Hydrogenophaga crassostreae* LPB0072 (Hydrog; unpublished; GenBank accession LVWD01000013), *Neisseria mucosa* C102 (N_muc; unpublished, GenBank accession GCA_000186165) and *Neisseria flavescens* SK114 (N_flav; unpublished; GenBank accession ACQV01000009). Signal peptide prediction was performed using SignalP software (Petersen et al., 2011). Signal peptide cleavage is predicted to occur at the conserved Ala residue. N-terminal and C-terminal domains and intervening linkers are indicated, based on the *X. citri* VirB9 cryo-EM structure within the core complex (Sgro et al., 2018). Alignments were performed using Clustal Omega (McWilliam et al., 2013) and Figures were produced by with JalView (Waterhouse et al., 2009).

Supp. Figure 10

Sgro et al. (2019)

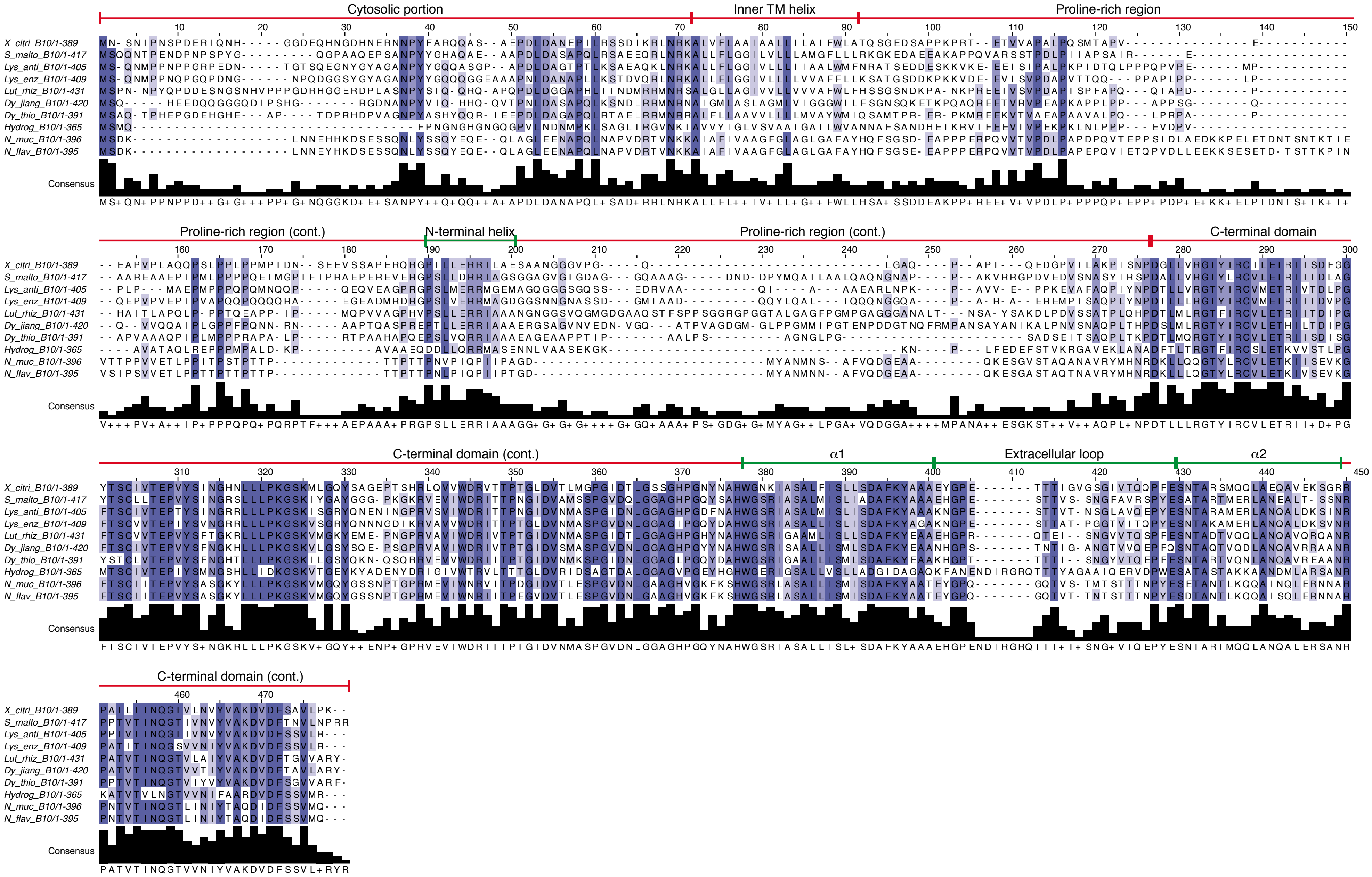

Supplementary Figure 10. X-T4SS VirB10 protein alignment. Multiple Sequence alignment of VirB10 proteins from the X-T4SSs of *Xanthomonas citri* 306 (X_citri; da Silva et al., 2002), *Stenotrophomonas maltophilia* K279a (S_malto; Crossman et al., 2008), *Lysobacter antibioticus* 76 (Lys_anti; de Bruijn et al., 2015), *Lysobacter enzymogenes* C3 (Lys_enz; unpublished; GenBank accession CP013140), *Luteibacter rhizovicinus* DSM16549 (Lut_rhiz; unpublished; GenBank accession CP017480), *Dyella jiangningensis* SBZ3-12 (Dy_jiang; Bao et al., 2014), *Dyella thiooxydans* ATSB10 (Dy_thio; unpublished; GenBank accession CP014841), *Hydrogenophaga crassostreae* LPB0072 (Hydrog; unpublished; GenBank accession LVWD01000013), *Neisseria mucosa* C102 (N_muc; unpublished, GenBank accession GCA_000186165) and *Neisseria flavescens* SK114 (N_flav; unpublished; GenBank accession ACQV01000009). General features mentioned in the text are indicated, based on the *X. citri* VirB10 cryo-EM structure within the core complex (Sgro et al., 2018): N-terminal cytosolic region, the hydrophobic transmembrane (TM) helix predicted to pass through the inner membrane, the proline-rich periplasmic portion of the N-terminal domain with an intervening helix that interacts with the VirB9 N-terminal domain, the C-terminal domain, and the helices (α1 and α2) and intervening loop that form the antenna and pore through the outer membrane. Transmembrane helix predictions were performed using the following algorithms: PredictProtein (Rost et al., 2007), HMMTop (Tusnády and Simon, 2001), TMpred (Hofmann and Stoffel, 1993), TMHMM (Krogh et al., 2001), TOPCONS (Tsirigos et al., 2015) and PSORT (Nakai and Horton, 1999). All predicted the N-terminal transmembrane (TM) helix for the X-T4SS VirB10 proteins. Alignments were performed using Clustal Omega (McWilliam et al., 2013) and Figures were produced with JalView (Waterhouse et al., 2009).

Supp. Figure 11

Sgro et al. (2019)

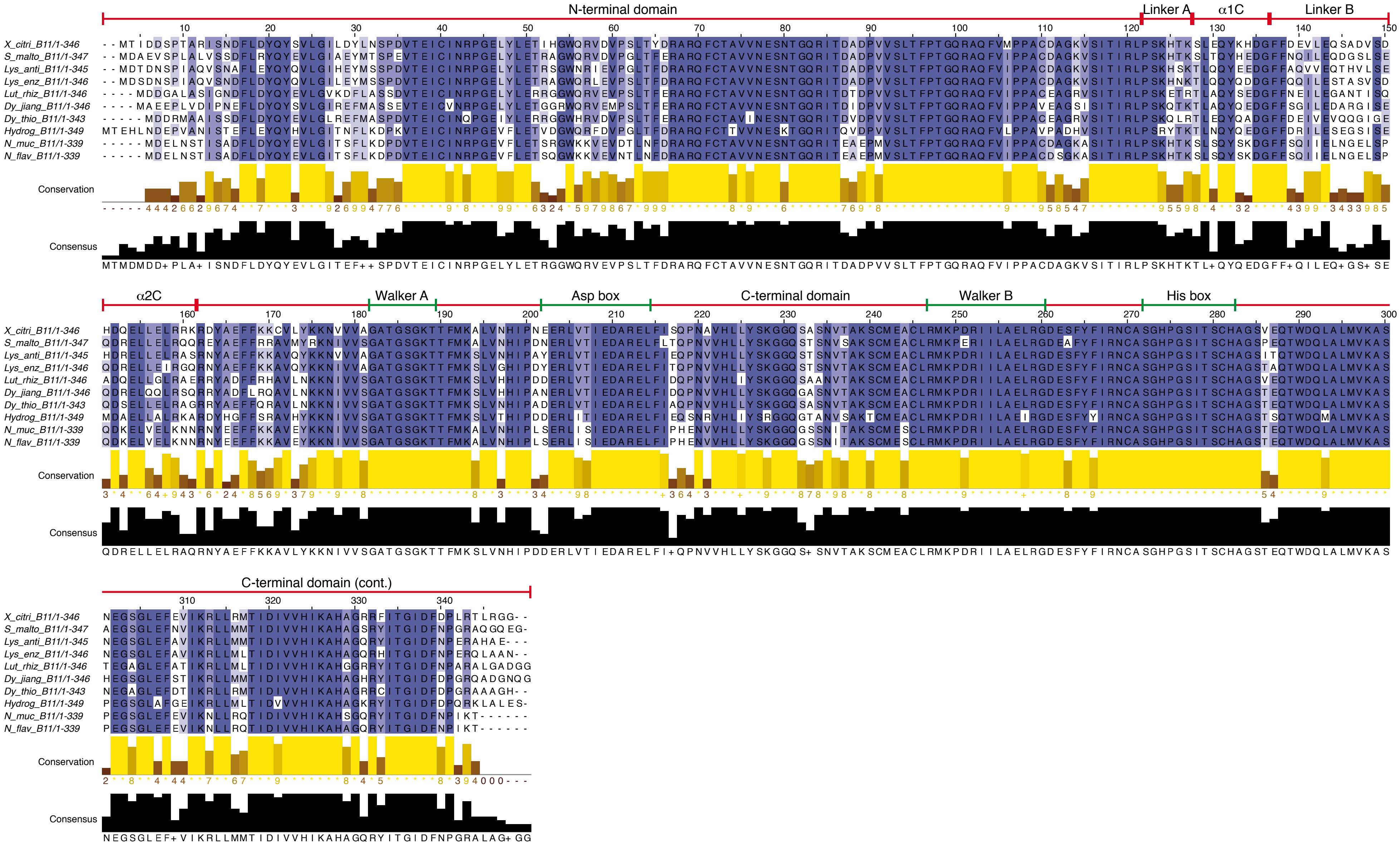

Supplementary Figure 11. X-T4SS VirB11 protein alignment. Multiple Sequence alignment of VirB11 proteins from the X-T4SSs of *Xanthomonas citri* 306 (X_citri; da Silva et al., 2002), *Stenotrophomonas maltophilia* K279a (S_malto; Crossman et al., 2008), *Lysobacter antibioticus* 76 (Lys_anti; de Bruijn et al., 2015), *Lysobacter enzymogenes* C3 (Lys_enz; unpublished; GenBank accession CP013140), *Luteibacter rhizovicinus* DSM16549 (Lut_rhiz; unpublished; GenBank accession CP017480), *Dyella jiangningensis* SBZ3-12 (Dy_jiang; Bao et al., 2014), *Dyella thiooxydans* ATSB10 (Dy_thio; unpublished; GenBank accession CP014841), *Hydrogenophaga crassostreae* LPB0072 (Hydrog; unpublished; GenBank accession LVWD01000013), *Neisseria mucosa* C102 (N_muc; unpublished, GenBank accession GCA_000186165) and *Neisseria flavescens* SK114 (N_flav; unpublished; GenBank accession ACQV01000009). General features mentioned in the text are indicated. Walker A, Walker B, Asp and His boxes involved in nucleotide binding are based on the correspondence between the sequence alignment and the analysis of VirB11 homologs in Krause et al. (2012). Features in the linker region between the N- and C-terminal domains were identified based on the analysis of Hare et al. (2006). Alignments were performed using Clustal Omega (McWilliam et al., 2013) and Figures were produced with JalView (Waterhouse et al., 2009).

Supp. Figure 12

Sgro et al. (2019)

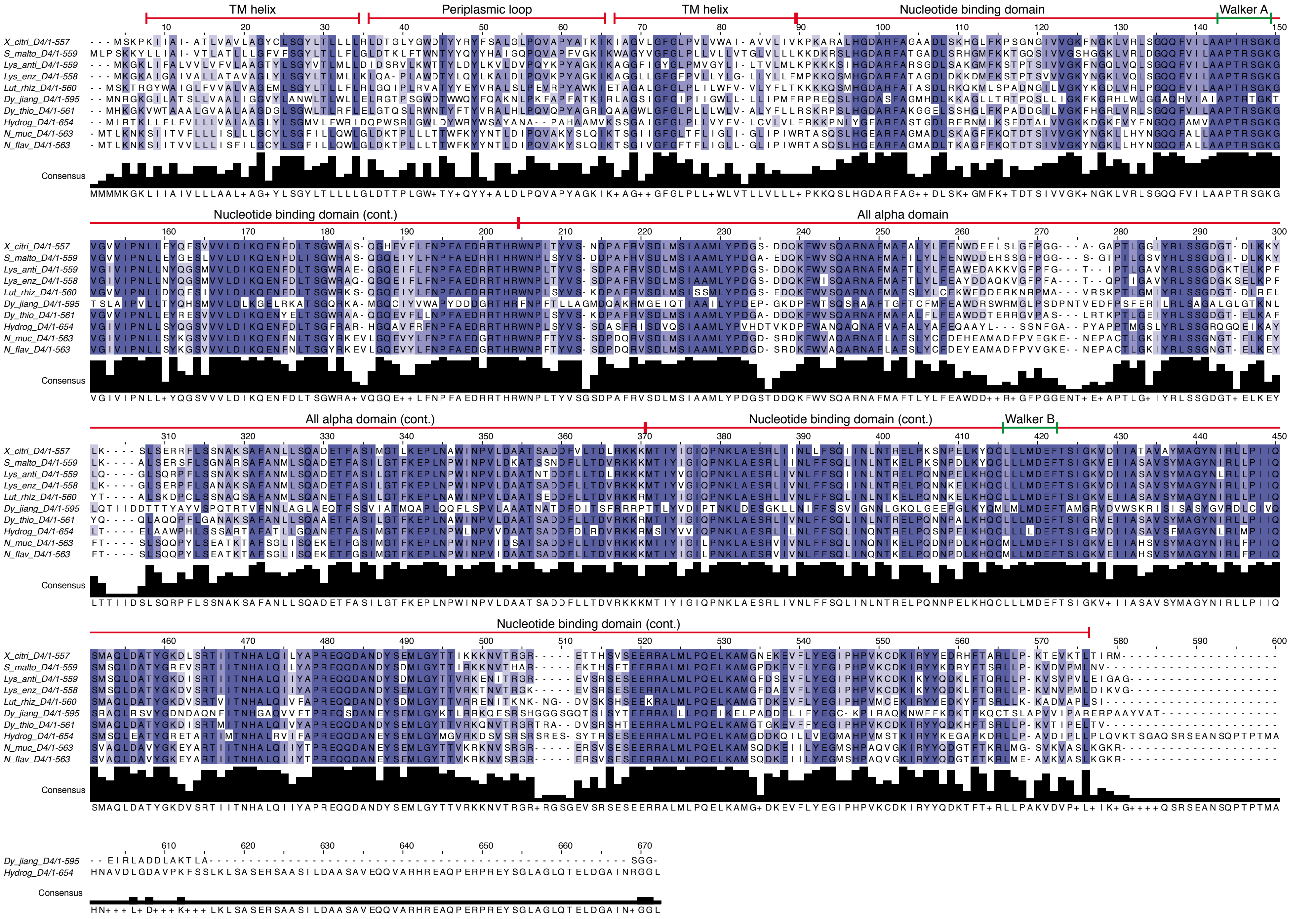

Supplementary Figure 12. X-T4SS VirD4 protein alignment. Multiple Sequence alignment of VirD4 proteins from the X-T4SSs of *Xanthomonas citri* 306 (X_citri; da Silva et al., 2002), *Stenotrophomonas maltophilia* K279a (S_malto; Crossman et al., 2008), *Lysobacter antibioticus* 76 (Lys_anti; de Bruijn et al., 2015), *Lysobacter enzymogenes* C3 (Lys_enz; unpublished; GenBank accession CP013140), *Luteibacter rhizovicinus* DSM16549 (Lut_rhiz; unpublished; GenBank accession CP017480), *Dyella jiangningensis* SBZ3-12 (Dy_jiang; Bao et al., 2014), *Dyella thiooxydans* ATSB10 (Dy_thio; unpublished; GenBank accession CP014841), *Hydrogenophaga crassostreae* LPB0072 (Hydrog; unpublished; GenBank accession LVWD01000013), *Neisseria mucosa* C102 (N_muc; unpublished, GenBank accession GCA_000186165) and *Neisseria flavescens* SK114 (N_flav; unpublished; GenBank accession ACQV01000009). The X-T4SS VirD4 proteins have a so-called canonical VirD4-like architecture (Llosa and Alkorta, 2017) with two N-terminal transmembrane helices separated by a ~30 amino acid periplasmic loop. The cytosolic C-terminal domain can be divided into a nucleotide binding domain, with conserved Walker A and Walker B motifs, and an intervening all-alpha domain. Domain and Walker A and B motif assignments were made with reference to the VirD4/TrwB structure (Gomis-Rüth et al., 2002). Transmembrane helix predictions were performed using the following algorithms: PredictProtein (Rost et al., 2007), HMMTop (Tusnády and Simon, 2001), TMpred (Hofmann and Stoffel, 1993), TMHMM (Krogh et al., 2001), TOPCONS (Tsirigos et al., 2015) and PSORT (Nakai and Horton, 1999). All predicted the two N-terminal transmembrane (TM) helices for the X-T4SS VirD4 proteins. Alignments were performed using Clustal Omega (McWilliam et al., 2013) and Figures were produced with JalView (Waterhouse et al., 2009).

Supplementary Figure References

Bao, Y., Kwok, A. H. Y., He, L., Jiang, J., Huang, Z., Leung, F. C. C., et al. (2014). Complete genome sequence of *Dyella jiangningensis* strain SBZ3-12, isolated from the surfaces of weathered rock. *Genome Announc.* 2. doi:10.1128/genomeA.00416-14.

Costa, T. R. D., Ilangovan, A., Ukleja, M., Redzej, A., Santini, J. M., Smith, T. K., et al. (2016). Structure of the bacterial sex F pilus reveals an assembly of a stoichiometric protein-phospholipid complex. *Cell* 166, 1436–1444.e10. doi:10.1016/j.cell.2016.08.025.

Crossman, L. C., Gould, V. C., Dow, J. M., Vernikos, G. S., Okazaki, A., Sebaihia, M., et al. (2008). The complete genome, comparative and functional analysis of Stenotrophomonas maltophilia reveals an organism heavily shielded by drug resistance determinants. *Genome Biol.* 9, R74. doi:10.1186/gb-2008-9-4-r74.

da Silva, A. C. R., Ferro, J. A., Reinach, F. C., Farah, C. S., Furlan, L. R., Quaggio, R. B., et al. (2002). Comparison of the genomes of two Xanthomonas pathogens with differing host specificities. *Nature* 417, 459–463. doi:10.1038/417459a.

de Bruijn, I., Cheng, X., de Jager, V., Expósito, R. G., Watrous, J., Patel, N., et al. (2015). Comparative genomics and metabolic profiling of the genus *Lysobacter*. *BMC Genomics* 16, 991. doi:10.1186/s12864-015-2191-z.

Gomis-Rüth, F. X., Moncalian, G., de la Cruz, F., Coll, M., Gomis-Ruth, F. X., Moncalian, G., et al. (2002). Conjugative plasmid protein TrwB, an integral membrane type IV secretion system coupling protein. *J. Biol. Chem.* 277, 7556–7566. doi:10.1074/jbc.M110462200 M110462200 [pii].

Hare, S., Bayliss, R., Baron, C., and Waksman, G. (2006). A large domain swap in the VirB11 ATPase of *Brucella suis* leaves the hexameric assembly intact. *J. Mol. Biol.* 360, 56–66. doi:10.1016/j.jmb.2006.04.060.

Hofmann, K., and Stoffel, W. (1993). TMbase - A database of membrane spanning proteins segments. *Biol. Chem. Hoppe. Seyler.* 374, 35.

Klausen, M. S., Jespersen, M. C., Nielsen, H., Jensen, K. K., Jurtz, V. I., Soenderby, C. K., et al. (2019). NetSurfP-2.0: Improved prediction of protein structural features by integrated deep learning. *Proteins Struct. Funct. Bioinforma.* 87, 520–527. doi:10.1002/prot.25674.

Krause, S., Bárcena, M., Pansegrau, W., Lurz, R., Carazo, J. M., and Lanka, E. (2012). Sequence-related protein export NTPases encoded by the conjugative transfer region of RP4 and by the *cag* pathogenicity island of *Helicobacter pylori* share similar hexameric ring structures. *Proc. Natl. Acad. Sci.* 97, 3067–3072. doi:10.1073/pnas.97.7.3067.

Krogh, A., Larsson, B., von Heijne, G., and Sonnhammer, E. L. L. (2001). Predicting transmembrane protein topology with a hidden Markov model: Application to complete genomes. *J. Mol. Biol.* 305, 567–580. doi:10.1006/jmbi.2000.4315.

Llosa, M., and Alkorta, I. (2017). “Coupling proteins in type IV secretion,” in *Current Topics in Microbiology and Immunology* (Springer, Cham), 143–168. doi:10.1007/978-3-319-75241-9_6.

McWilliam, H., Li, W., Uludag, M., Squizzato, S., Park, Y. M., Buso, N., et al. (2013). Analysis tool web services from the EMBL-EBI. *Nucleic Acids Res.* 41, W597–W600. doi:10.1093/nar/gkt376.

Nakai, K., and Horton, P. (1999). PSORT: A program for detecting sorting signals in proteins and predicting their subcellular localization. *Trends Biochem. Sci.* 24, 34–35. doi:10.1016/S0968-0004(98)01336-X.

Oliveira, L. C., Souza, D. P., Oka, G. U., Lima, F. da S., Oliveira, R. J., Favaro, D. C., et al. (2016). VirB7 and VirB9 interactions are required for the assembly and antibacterial activity of a type IV secretion system. *Structure* 24, 1707–1718. doi:10.1016/j.str.2016.07.015.

Petersen, T. N., Brunak, S., Von Heijne, G., and Nielsen, H. (2011). SignalP 4.0: Discriminating signal peptides from transmembrane regions. *Nat. Methods* 8, 785–786. doi:10.1038/nmeth.1701.

Rost, B., Yachdav, G., and Liu, J. (2007). The PredictProtein server. *Nucleic Acids Res.* 32, W321–W326. doi:10.1093/nar/gkh377.

Sgro, G. G., Costa, T. R. D., Cenens, W., Souza, D. P., Cassago, A., Coutinho de Oliveira, L., et al. (2018). Cryo-EM structure of the bacteria-killing type IV secretion system core complex from *Xanthomonas citri*. *Nat. Microbiol.* 3, 1. doi:10.1038/s41564-018-0262-z.

Souza, D. P., Andrade, M. O., Alvarez-Martinez, C. E., Arantes, G. M., Farah, C. S., and Salinas, R. K. (2011). A component of the *Xanthomonadaceae* type IV secretion system combines a VirB7 motif with a N0 domain found in outer membrane transport proteins. *PLoS Pathog.* 7, e1002031. doi:10.1371/journal.ppat.1002031.

Tsirigos, K. D., Peters, C., Shu, N., Käll, L., and Elofsson, A. (2015). The TOPCONS web server for

consensus prediction of membrane protein topology and signal peptides. *Nucleic Acids Res.* 43, W401–W407. doi:10.1093/nar/gkv485.

Tusnády, G. E., and Simon, I. (2001). The HMMTOP transmembrane topology prediction server. *Bioinformatics* 17, 849–850. doi:10.1093/bioinformatics/17.9.849.

Wallden, K., Williams, R., Yan, J., Lian, P. W., Wang, L., Thalassinos, K., et al. (2012). Structure of the VirB4 ATPase, alone and bound to the core complex of a type IV secretion system. *Proc. Natl. Acad. Sci.* 109, 11348–11353. doi:10.1073/pnas.1201428109.

Waterhouse, A. M., Procter, J. B., Martin, D. M. A., Clamp, M., and Barton, G. J. (2009). Jalview Version 2-A multiple sequence alignment editor and analysis workbench. *Bioinformatics* 25, 1189–1191. doi:10.1093/bioinformatics/btp033.

Zupan, J., Hackworth, C. A., Aguilar, J., Ward, D., and Zambryski, P. (2007). VirB1* promotes T-pilus formation in the *vir*-type IV secretion system of *Agrobacterium tumefaciens*. *J. Bacteriol.* 189, 6551–6563. doi:10.1128/JB.00480-07.
